# Efficacy of Active vs Sham Intermittent Theta Burst Transcranial Magnetic Stimulation for Patients With Bipolar Depression: A Randomized Clinical Trial

**DOI:** 10.1001/jamanetworkopen.2021.0963

**Published:** 2021-03-12

**Authors:** Alexander McGirr, Fidel Vila-Rodriguez, Jaeden Cole, Ivan J. Torres, Shyam Sundar Arumugham, Kamyar Keramatian, Gayatri Saraf, Raymond W. Lam, Trisha Chakrabarty, Lakshmi N. Yatham

**Affiliations:** 1 Department of Psychiatry, University of Calgary, Alberta, Canada; 2 Hotchkiss Brain Institute, University of Calgary, Calgary, Alberta, Canada; 3 Mathison Centre for Mental Health Research and Education, Calgary, Alberta, Canada; 4 Non-Invasive Neurostimulation Therapies Laboratory, Department of Psychiatry, The University of British Columbia, Vancouver, British Columbia, Canada; 5 Department of Psychiatry, University of British Columbia, Vancouver, British Columbia, Canada; 6 BC Mental Health and Substance Use Services, Vancouver, British Columbia, Canada; 7 Department of Psychiatry, National Institute of Mental Health and Neurosciences, India

## Abstract

**Importance:**

Major depressive episodes in bipolar disorder are common and debilitating. Repetitive transcranial magnetic stimulation is well established in the treatment of major depressive disorder, and the intermittent theta burst stimulation (iTBS) protocol is replacing conventional protocols because of noninferiority and reduced delivery time. However, iTBS has not been adequately studied in bipolar disorder and, therefore, its efficacy is uncertain.

**Objective:**

To determine whether iTBS to the left dorsolateral prefrontal cortex (LDLPFC) is safe and efficacious in the treatment of acute bipolar depression.

**Design, Setting, and Participants:**

This study was a double-blind, 4-week, randomized clinical trial of iTBS targeting the LDLPFC. Two Canadian academic centers recruited patients between 2016 and 2020. Adults with bipolar disorder type I or type II experiencing an acute major depressive episode were eligible if they had not benefited from a first-line treatment for acute bipolar depression recommended by the Canadian Network for Mood and Anxiety Treatments and were currently treated with a mood stabilizer, an atypical antipsychotic, or their combination. Seventy-one participants were assessed for eligibility, and 37 were randomized to daily sham iTBS or active iTBS using a random number sequence, stratified according to current pharmacotherapy. Data analysis was performed from April to September 2020.

**Interventions:**

Four weeks of daily active iTBS (120% resting motor threshold) or sham iTBS to the LDLPFC. Nonresponders were eligible for 4 weeks of open-label iTBS.

**Main Outcomes and Measures:**

The primary outcome was the change in score on the Montgomery-Asberg Depression Rating Scale from baseline to study end. Secondary outcomes included clinical response, remission, and treatment-emergent mania or hypomania.

**Results:**

The trial was terminated for futility after 37 participants (23 women [62%]; mean [SD] age, 43.86 [13.87] years; age range, 20-68 years) were randomized, 19 to sham iTBS and 18 to active iTBS. There were no significant differences in Montgomery-Asberg Depression Rating Scale score changes (least squares mean difference between groups, −1.36 [95% CI, −8.92 to 6.19; *P* = .91] in favor of sham iTBS), and rates of clinical response were low in both the double-blind phase (3 of 19 participants [15.8%] in the sham iTBS group and 3 of 18 participants [16.7%] in the active iTBS group) and open-label phase (5 of 21 participants [23.8%]). One active iTBS participant had a treatment emergent hypomania, and a second episode occurred during open-label treatment.

**Conclusions and Relevance:**

iTBS targeting the LDLPFC is not efficacious in the treatment of acute bipolar depression in patients receiving antimanic or mood stabilizing agents. Additional research is required to understand how transcranial magnetic stimulation treatment protocols differ in efficacy between unipolar and bipolar depression.

**Trial Registration:**

ClinicalTrials.gov Identifier: NCT02749006

## Key Points

**Question:**

Is intermittent theta burst stimulation (iTBS) transcranial magnetic stimulation targeting the left dorsolateral prefrontal cortex safe and efficacious in acute bipolar depression?

**Findings:**

This randomized clinical trial including 37 adults was terminated for futility. There was no evidence of antidepressant superiority for active iTBS over sham iTBS, and safety is uncertain because 1 hypomanic switch occurred with active iTBS and a second occurred during the open-label phase.

**Meaning:**

iTBS targeting the left dorsolateral prefrontal cortex is not efficacious in the treatment of acute bipolar depression and can result in mood episode switches despite treatment with a mood stabilizer or antipsychotic medication.

## Teaser

This randomized clinical trial examines whether intermittent theta burst transcranial magnetic stimulation (TMS) to the left dorsolateral prefrontal cortex is safe and efficacious in the treatment of acute bipolar depression.

## Introduction

Bipolar disorder (BD) is a common lifetime condition that affects up to 2% of the population.^1,2,3^ Although episodes of mania and hypomania are defining features of the disorder, syndromal and subsyndromal depressive symptoms account for the major disease burden and a substantial proportion of disability.^4,5^ Patients are reported to spend as much as half of their lives with mood symptoms, with depressive symptoms accounting for 70% to 82% of the symptomatic periods.^6^ The resulting functional and role impairment is significant.^7^

There are several US Food and Drug Administration (FDA)–approved medications with level 1 evidence for efficacy in the management of acute mania. However, to date, only 4 treatments with level 1 or level 2 evidence (ie, olanzapine and fluoxetine combination, quetiapine, lurasidone, and cariprazine) have been approved by the FDA for treatment of acute bipolar depression.^8^ Moreover, many patients with bipolar depression either do not respond or have difficulty tolerating the adverse effects of these medications. Therefore, novel treatments are urgently needed for management of acute bipolar depression to address this unmet clinical need.

Repetitive transcranial magnetic stimulation (rTMS) therapy is a noninvasive neurostimulation treatment that has been adopted as a first-line treatment for pharmacotherapy-resistant major depressive disorder (MDD).^9^ rTMS induces electrical activity in the cortex using magnetic fields generated outside of the head. Repetitive magnetic pulses delivered using high-frequency, low-frequency, bilateral, priming, and theta burst protocols have demonstrated efficacy in improving depression in MDD.^10^ However, the evidence for antidepressant efficacy of rTMS in the treatment of bipolar depression is limited and derived primarily from small trials^11,12,13,14,15^ and subsets of trials in major depression that included individuals with BD.^16,17^

Newer TMS protocols involving theta burst stimulation have garnered significant research attention and evidence for antidepressant efficacy.^18^ In particular, intermittent theta burst stimulation (iTBS) is a protocol that produces lasting neurophysiological changes,^19,20^ has demonstrated antidepressant efficacy in MDD,^21^ and has recently been shown to be noninferior to high-frequency stimulation in MDD in a large single-blind study.^22^ This is important, because iTBS is a short protocol that could result in a drastic shift in the cost curve of this treatment. Yet, high-frequency stimulation and iTBS are not physiologically equivalent,^23^ and, therefore, they may not have comparable efficacy in different clinical populations. Indeed, despite the growing evidence base supporting iTBS in the treatment of MDD, antidepressant efficacy for iTBS has yet to be demonstrated in bipolar depression. Limited data to date do not support the antidepressant efficacy of iTBS in a pilot randomized clinical trial (RCT) of twice-daily iTBS compared with sham in patients with bipolar depression^24^ or for subsets of patients with bipolar depression who are included in larger studies with once daily treatments.^16^

In real-world clinical practice, rTMS is typically offered after a patient does not respond to or has trouble tolerating at least 1 of the first-line treatments. To mirror clinical practice, in this study, we examined the antidepressant efficacy of iTBS in patients with bipolar depression who did not respond to or had trouble tolerating at least 1 of the first-line treatments recommended by the Canadian Network for Mood and Anxiety Treatments (CANMAT) and International Society for Bipolar Disorders (ISBD)^8^ using a randomized, sham-controlled, double-blind design.

## Methods

We conducted a 4-week, double-blind RCT in 2 Canadian centers (University of British Columbia [UBC], British Columbia, Canada; and University of Calgary [UC], Alberta, Canada). Data were collected between October 2016 and March 2020. The study was approved by the Clinical Research Ethics Board of the UBC and the Conjoint Health Research Ethics Board of the UC. Participants provided written informed consent. We followed the Consolidated Standards of Reporting Trials (CONSORT) reporting guideline for RCTs.

### Participants

Participants were recruited by referral, as well as online and community advertisements. Eligibility criteria were men and women aged 18 to 70 years with a primary diagnosis of BD type I or type II by *Diagnostic and Statistical Manual of Mental Disorders* (Fifth Edition) criteria, experiencing a major depressive episode (MDE) with a score of 18 or higher on the 17-item Hamilton Depression Rating Scale,^25^ and no clinical response to at least 1 CANMAT-recommended first-line treatment for an acute MDE of BD (lithium, lamotrigine, quetiapine, lurasidone with or without concurrent lithium, or valproate). Participants were required to have been taking a stable pharmacological regimen for 2 weeks before screening that had to include a mood stabilizer (lithium >0.6 mEq/L or valproate >350 mM/L), an atypical antipsychotic, or a combination of a mood stabilizer and an atypical antipsychotic. For participants with BD type II, lamotrigine monotherapy was acceptable if the dose was greater than 100 mg daily. Patients who had been taking antidepressant medications (2 patients took bupropion, and 1 each took escitalopram, mirtazapine, and venlafaxine) before the enrollment were allowed to continue these at same dose levels during the double-blind phase of the study. Exclusion criteria were acute suicidality, current psychosis, a substance use disorder within the last 3 months, history of seizures, pacemaker or metallic implant, unstable medical condition, and comorbid psychiatric conditions that were deemed to be primary. Other exclusion criteria included previous nonresponse to rTMS, current use of more than 3 antipsychotic agents, nonresponse to electroconvulsive therapy in the current episode, and psychotherapy initiated within the last 3 months.

### Protocol and Randomization

The study protocol is available in Supplement 1. An independent statistician generated a stratified random number sequence for each site for participants treated with mood stabilizers, atypical antipsychotics, or their combination. Eligible patients were randomized with allocation concealment via the envelope method and remained blind to their treatment condition throughout the duration of the study.

Both sites used a MagPro X100 stimulator (MagVenture), with the UBC site using a dual active-sham Cool-B65 A/P coil and the UC site using a COOL-B70 or MCF-P-B70 placebo coil (both from MagVenture). Both did so in conjunction with participant anatomical magnetic resonance imaging and neuronavigation (Visor2, ANT Neuro). Resting motor threshold was determined by visual inspection at the UBC site and by using electromyographic electrodes at the UC site placed over the first dorsal interosseous muscle, with threshold determined as the stimulus intensity required to elicit 5 of 10 electromyographic responses greater than 50 μV.

Patients were randomly allocated to either sham or active iTBS, consisting of a total of 600 pulses per session delivered as triplets at 50 Hz repeated at 5 Hz (2 seconds on and 8 seconds off) at 120% resting motor threshold. These stimulus parameters have been shown to be noninferior to evidence-based high-frequency stimulation in improving depressive symptoms in patients with MDD in a large single-blind study.^22^ We targeted the left dorsolateral prefrontal cortex (LDLPFC) using neuronavigation, and the BeamF3 method^26^ for 1 participant who could not tolerate undergoing magnetic resonance imaging. Participants received treatments daily Monday through Friday for a total of 20 treatments. At the conclusion of the double-blind phase, participants who did not exhibit a 50% or more reduction in Montgomery-Asberg Depression Rating Scale (MADRS)^27^ score were offered an additional 4 weeks of open-label iTBS.

### Assessments

Sociodemographic information, including sex and ethnicity, were self-reported by participants. Participants were assessed by independent evaluators blind to treatment condition. The diagnosis of BD type I or II with an MDE was confirmed with the Mini International Neuropsychiatric Interview 7.0. The 17-item Hamilton Depression Rating Scale was administered for screening purposes, and participants were deemed eligible if their score was 18 or higher. The primary outcome of the study was change in depressive symptoms assessed using the MADRS from baseline to end point; MADRS was administered at baseline, 2 weeks, and 4 weeks or end point.

Secondary outcomes included rates of clinical response, defined as a reduction of 50% or more in MADRS score, and clinical remission, defined as a MADRS score of 12 or less at the end point. The Young Mania Rating Scale (YMRS)^28^ was used for capturing treatment emergent mood switches, which were defined as a YMRS score of 12 or higher. Clinicians also administered the Clinical Global Impression^29^ subscales to assess overall illness severity and improvement. A patient’s perception of illness was evaluated with the Patient Global Impression Rating Scale, a visual analog scale for overall well-being (range, 0-100 points), and the Brief Illness Perception Questionnaire^30^ at baseline and after 4 weeks. Functioning was assessed using the Sheehan Disability Scale^31^; participants rated impairment in work and school, social, and family life at baseline and after 4 weeks. BD-specific quality of life was assessed using the Quality of Life in Bipolar Disorder scale.^32^ Cognitive function was assessed at baseline and after 4 weeks using self-reported measures and neuropsychological testing, and these data will be reported elsewhere.

Self-reported depressive symptoms were measured using the Quick Inventory of Depressive Symptomatology-Self Report,^33^ and anxiety symptoms were measured using the Generalized Anxiety Disorder 7-item scale.^34^ At the conclusion of the blinded study, participants were asked whether they believed they received active or sham iTBS. Adverse events were recorded. During the open-label phase of the trial, clinician-administered and self-reported scales were repeated at weeks 6 and 8.

### Statistical Analysis

Statistical analyses were performed with SPSS statistical software version 26 (IBM). The target sample size of 50 participants per condition would have allowed 80% power to detect an effect size of 0.3 or higher with α ≤ .05. However, because of slow recruitment and very low overall response rates, an independent statistician was asked to conduct an interim futility analysis with a conditional power of 20% or higher. This criterion was not met, and, therefore, the study was terminated for futility.

We used 2-sided *t* test for continuous variables and Fisher exact test or χ^2^ test for categorical variables. The assumption of normality for continuous variables was tested using the Shapiro-Wilks test. To analyze the primary outcome and continuous secondary outcomes, we used linear mixed-effect models with restricted maximum likelihood estimation. In these models, repeated measures of clinical symptoms were the dependent variable, group status was the fixed-effect variable, and a random effect was used for site. The group-by-time interaction is reported for these models. We also performed analyses examining the effect of primary diagnosis (BD type I vs BD type II). Significance was set at α ≤ .05. Results are reported as mean (SD) or mean (95% CI). Data analysis was performed from April to September 2020.

## Results

**Figure 1.**
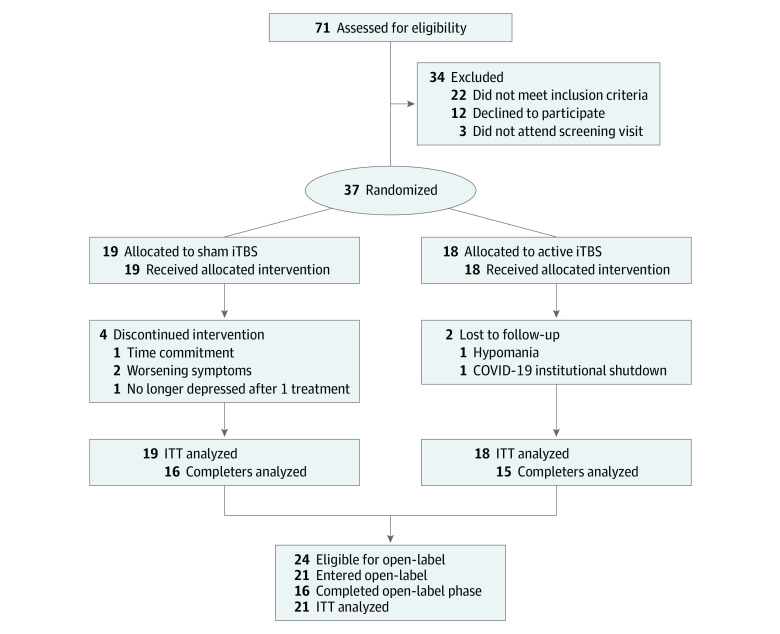
Participant Enrollment Flowchart COVID-19 indicates coronavirus disease 2019; iTBS, intermittent theta burst stimulation; ITT, intention to treat.

Participant flow is illustrated in Figure 1. Of a total of 71 participants screened, 37 participants (23 women [62%]; mean [SD] age, 43.86 [13.87] years; age range, 20-68 years) were eligible and were randomized to treatment (19 to sham iTBS and 18 to active iTBS). There were no demographic or clinical variables that differentiated the active and sham groups (Table 1). The sham and active iTBS groups both initially presented with moderate-severity depression (mean [SD] MADRS scores, 32.57 [4.00] for sham iTBS vs 33.38 [4.46] for active iTBS) and were not exhibiting manic or hypomanic symptoms (mean [SD] YMRS scores, 2.05 [1.68] for sham iTBS vs 2.11 [1.41] for active iTBS). Inclusion depended on not responding to at least 1 CANMAT-ISBD level 1 recommended treatment for the management of acute bipolar depression, and there were no differences in number of previously failed first-line treatments between the 2 groups (mean [SD], 1.42 [0.90] treatments for sham iTBS vs 1.72 [1.17] treatments for active iTBS). Stratified randomization achieved comparable distributions of participants treated with mood stabilizer monotherapy, atypical antipsychotic monotherapy, and combination mood stabilizer and atypical antipsychotic.

**Table 1.** Clinical and Demographic Characteristics of the Randomized Participants

Characteristic	Participants, No. (%)		
	Full sample (N = 37)	Sham iTBS (n = 19)	Active iTBS (n = 18)
Age, mean (SD), y	43.86 (13.87)	43.00 (14.34)	44.78 (13.71)
Female	23 (62.2)	12 (63.2)	11 (61.1)
Race/ethnicity			
Asian	2 (5.4)	1 (5.3)	1 (5.6)
White	34 (91.9)	17 (89.5)	17 (94.4)
Middle Eastern	1 (2.7)	1 (5.3)	0 (0.0)
Married or common law spouse	15 (40.5)	5 (26.4)	10 (55.5)
Duration of education, mean (SD), y	15.32 (3.09)	14.95 (2.17)	15.72 (3.86)
Employed	15 (40.5)	7 (36.8)	8 (44.4)
Right handed	32 (86.5)	17 (89.5)	15 (83.3)
Primary diagnosis			
Bipolar disorder type I	21 (56.8)	10 (52.6)	11 (61.1)
Bipolar disorder type II	16 (43.3)	9 (47.4)	7 (38.9)
Duration of current episode, mean (SD), wk	32.45 (34.29)	31.88 (33.19)	33.00 (36.31)
Hamilton Rating Scale for Depression score, mean (SD)	22.10 (3.84)	21.63 (4.11)	22.61 (3.58)
Montgomery Asberg Depression Rating Scale score, mean (SD)	32.97 (4.19)	32.57 (4.00)	33.38 (4.46)
Young Mania Rating Scale score, mean (SD)	2.29 (1.79)	2.11 (1.41)	2.05 (1.68)
Clinical Global Impression–Severity score, mean (SD)	4.62 (0.73)	4.47 (0.77)	4.65 (0.86)
Failed Canadian Network for Mood and Anxiety Treatments level 1 treatments for acute bipolar depression (current), mean (SD), No.	1.56 (1.04)	1.42 (0.90)	1.72 (1.17)
Medication stratification			
Mood stabilizer	16 (43.2)	8 (42.1)	8 (44.4)
Atypical antipsychotic	3 (8.1)	2 (10.5)	1 (5.6)
Mood stabilizer and atypical antipsychotic	18 (48.6)	9 (47.4)	9 (50.0)
Site			
University of British Columbia	16 (43.2)	9 (56.3)	7 (43.8)
University of Calgary	21 (56.8)	10 (47.6)	11 (42.4)

Abbreviation: iTBS, intermittent theta burst stimulation.

### Double-Blind Phase Results

Two participants receiving active iTBS and 4 participants receiving sham iTBS dropped out of the study. One participant receiving active iTBS completed 3 weeks of treatment before a coronavirus pandemic–related institutional closure prematurely ended treatment. One treatment-emergent mood switch occurred in a participant allocated to active iTBS after the first treatment session.

Blinding integrity was preserved, with 8 of 16 active iTBS participants and 13 of 17 sham iTBS participants who completed the study correctly guessing their allocation at the conclusion of treatment (*P* = .15, Fisher exact test). Change in depressive symptoms over the course of the trial was significantly associated with whether participants believed they received active or sham iTBS (mean [SD] percentage MADRS score decrease, 47.47% [30.44%] vs 14.84% [22.03%]; median [interquartile range], 44.59% [31.84%-71.61%] vs 10.09% [1.76%-26.85%]; *t*_28_ = 3.41; *P* = .002). More participants at the UBC site believed they were receiving sham iTBS than at the UC site (86.7% vs 44.4%; *P* = .03, Fisher exact test).

**Figure 2.**
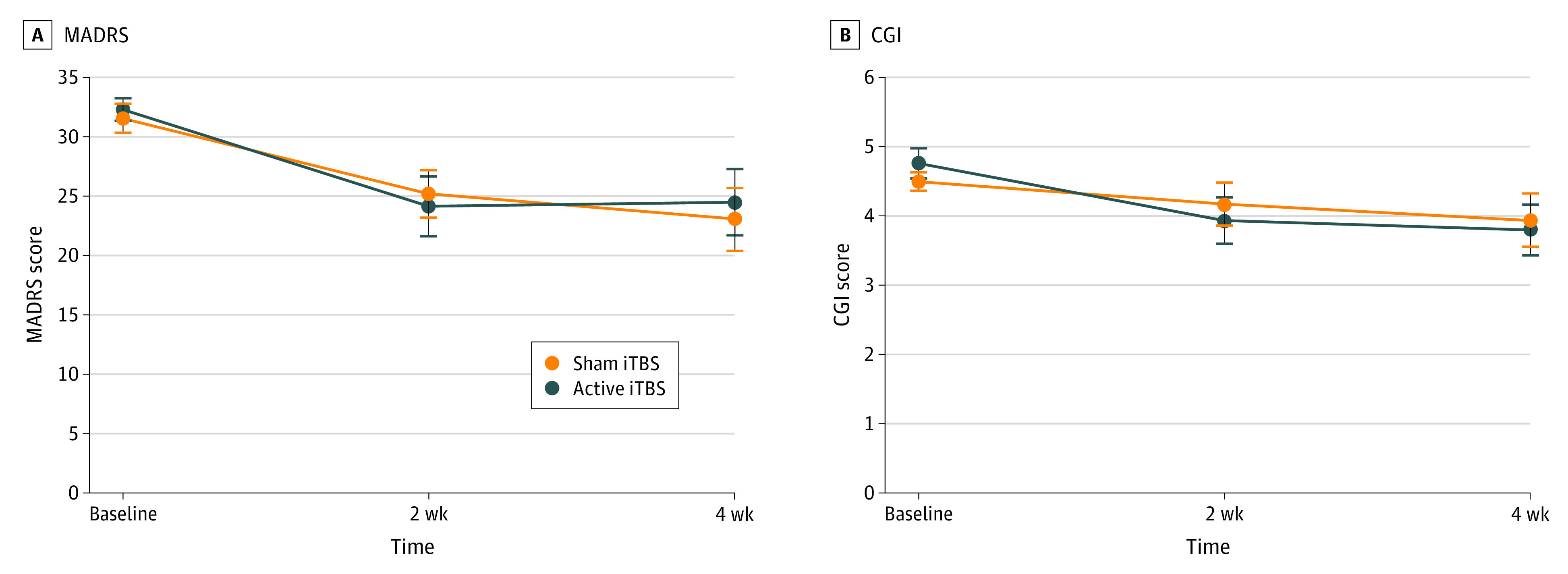
Clinical Outcomes in Patients Who Received Sham and Active Intermittent Theta Burst Stimulation (iTBS) Panel A shows the change in Montgomery-Asberg Depression Rating Scale (MADRS) score from baseline to study end at 4 weeks. Panel B shows the change in scores on the Clinical Global Impression (CGI) Scale. Circles denote means, and error bars denote standard error of the mean.

Clinical outcomes of the double-blind phase are detailed in Table 2. Repeated-measures linear mixed-effect models found no evidence for clinical superiority of active iTBS, as indicated by no significant difference in MADRS change scores between the 2 groups (Figure 2A). The least squares mean difference for MADRS scores at week 4 was –1.36 (95% CI, –8.92 to 6.19; *P* = .91) in favor of sham iTBS. The generalized linear mixed-effect model was repeated to test for possible efficacy differences between BDI and BDII. We saw no evidence of differential efficacy (BD type I vs BD type II by group ty time, *F*_1,61.33_ = 1.12; *P* = .29). We did not observe any differences in improvement according to different mood stabilizers (full sample, 4 participants taking lamotrigine [*t*_29_ = 0.34; *P* = .73], 15 participants taking lithium [*t*_29_ = 0.33; *P* = .74], and 8 participants taking valproate [*t*_29_ = 0.38; *P* = .63]; active iTBS sample, 2 participants taking lamotrigine [*t*_13_ = 0.47; *P* = .64], 8 participants taking lithium [*t*_13_ = 0.69; *P* = .49], and 4 participants taking valproate [*t*_13_ = 0.54; *P* = .59]).

**Table 2.** Study Outcome Measures

Outcome measure	Sham iTBS		Active iTBS		Least squares mean difference, mean difference or odds ratio (95% CI)	Group-by-time interaction, Fisher exact or *t *test	*P* value
	Participants, No.	Score, mean (SD)	Participants, No.	Score, mean (SD)			
Primary outcome: MADRS score							
Baseline	19	31.52 (5.22)	18	32.27 (4.04)	–1.36 (–8.92 to 6.19)	*F*_1,63.50_, 0.01	.91
2 wk	17	25.17 (8.24)	16	24.12 (10.09)			
4 wk	16	23.06 (10.58)	15	24.46 (10.82)			
Secondary outcomes, No. of participants/total No.							
Clinical response (MADRS score decrease by ≥50%)	3/19		3/18		0.93 (0.16 to 5.38)	NA	.94
Clinical remission (MADRS score ≤12)	3/19		3/18				
Young Mania Rating Scale							
Baseline	19	2.05 (1.68)	18	2.11 (1.41)	1.10 (0.11 to 2.09)	*F*_1,63.83_, 2.50	.11
2 wk	17	1.94 (1.91)	16	1.50 (1.89)			
4 wk	16	2.25 (1.23)	15	1.13 (1.55)			
Treatment-emergent mania or hypomania, No. of participants/total No.	0/19		1/18		NA	NA	.29
Clinical Global Impression-Severity subscale							
Baseline	19	4.50 (0.51)	18	4.76 (0.90)	–0.15 (–0.86 to 1.17)	*F*_1,65.30_, 1.10	.29
2 wk	16	4.17 (1.28)	16	3.93 (1.34)			
4 wk	16	3.93 (1.52)	15	3.80 (1.42)			
Clinical Global Impression-Improvement subscale		2.94 (1.23)		3.00 (1.30)	0.06 (–0.99 to 0.87)	*t*_29_ , 0.13	.89
Patient global impression rating scale-severity							
Baseline	18	3.05 (0.80)	18	3.16 (0.51)	–0.07 (–0.67 to 0.51)	*F*_1,58.39_, 0.34	.56
4 wk	15	2.66 (0.81)	15	2.60 (0.82)			
Patient global impression rating scale-improvement	15	3.20 (1.26)	15	3.53 (1.24)	0.33 (–0.60 to 1.27)	*t*_28_, 0.72	
Overall well-being (visual analog scale)							
Baseline	19	35.21 (18.80)		41.83 (18.29)	11.66 (–1.25 to 24.57)	*F*_1,65.58_, 0.37	.54
4 wk	15	46.66 (18.09)		58.13 (17.13)			
Brief Illness Perception Questionnaire							
Baseline	18	59.13 (8.21)	17	60.35 (4.67)	4.47 (–1.36 to 10.32)	*F*_1,60.55_, 0.57	.45
4 wk	15	58.93 (8.37)	16	57.31 (8.27)			
Sheehan Disability Scale							
Baseline	19	23.44 (6.43)	18	23.83 (5.09)	0.10 (–6.64 to 6.85)	*F*_1,49.78_, 0.013	.90
4 wk	15	19.23 (8.26)	16	19.40 (10.32)			
Quality of Life in Bipolar Disorder							
Baseline	19	108.78 (26.34)	18	109.00 (18.67)	5.20 (–20.64 to 31.05)	*F*_1,47.75_, 0.16	.69
4 wk	13	130.76 (36.54)	16	136.18 (33.81)			
Participant self-reported symptoms							
Quick Inventory of Depressive Symptoms–Self-Rated							
Baseline	19	19.77 (4.82)	18	21.29 (6.16)	–1.21 (–6.62 to 4.19)	*F*_1,59.16_, 1.24	.26
2 wk	16	16.18 (5.41)	16	12.75 (6.91)			
4 wk	15	15.80 (8.05)	14	14.85 (6.81)			
Generalized Anxiety Disorder 7-item							
Baseline	19	12.52 (4.94)	18	12.38 (5.86)	–0.61 (–4.86 to 3.63)	*F*_1,62.24_, 0.11	.73
2 wk	16	10.81 (3.60)	16	8.06 (6.06)			
4 wk	15	9.13 (5.59)	15	8.66 (6.34)			

Abbreviations: MADRS, Montgomery Asberg Depression Rating Scale; NA, not applicable.

Similarly, there were no significant differences on any of the secondary outcomes between active iTBS and sham iTBS (see Table 2 for details; Figure 2B shows Clinical Global Impression scores). In particular, there were no differences in rates of clinical response at the conclusion of the double-blind phase (3 of 19 participants [15.8%] in the sham iTBS group vs 3 of 18 participants [16.7%] in the active iTBS group responded to treatment; *P* > .99, Fisher exact test), and all participants who achieved clinical response also achieved clinical remission.

Change in self-reported depressive symptoms (Quick Inventory of Depressive Symptomatology-Self Report) and anxiety symptoms (Generalized Anxiety Disorder 7-item scale) similarly did not differ between groups (Table 2). No seizures occurred. No other substantial adverse events occurred.

### Open-Label Phase Results

Twenty-four of the 29 participants who completed the double-blind phase of the trial were eligible to continue with the open-label phase. Three participants declined to continue with open-label treatment because of a perceived lack of effectiveness and time constraints.

Of the 21 participants who entered the open-label phase, 16 completed 4 weeks of open-label iTBS treatment. One participant who had been receiving sham iTBS during the double-blind phase had a treatment-emergent hypomania after the sixth open-label treatment. Three participants withdrew during the open-label phase, and coronavirus-related institutional closure prematurely terminated open-label treatment for 1 participant after 3 weeks.

From the end of the double-blind phase, 5 of 21 open-label participants (23.8%) achieved clinical response and 4 of 21 (19.0%) achieved clinical remission. Three of the 5 clinical responders had been allocated to the sham iTBS condition during the double-blind phase.

## Discussion

This 2-site, double-blind, sham-controlled RCT of iTBS in treating MDEs of BD among participants who were resistant or intolerant to at least 1 first-line treatment was halted for futility. We found no evidence for antidepressant efficacy of iTBS using clinician-rated depressive symptoms and no differences in any of the secondary outcomes. Indeed, the improvement in the MADRS score was greater in the sham group but the magnitude of difference was very small and is below the minimally clinically important difference.^35^ Although manic and hypomanic symptoms did not differ between groups, the only treatment-emergent mood switch during the double-blind phase occurred after a single session of active iTBS and a second occurred during the open-label phase.

On the basis of efficacy data from samples including both BD and MDD, rTMS was approved by the FDA and Health Canada for the treatment of major depressive episodes regardless of primary diagnosis. Nevertheless, the randomized, blinded, sham-controlled literature using TMS in bipolar depression is limited,^16,17^ and there has been a dearth of research dedicated to how specific protocols differ in their clinical efficacy between BD and MDD. Although there is preliminary evidence for efficacy of high-frequency rTMS,^16,17^ low-frequency rTMS,^16,17^ and high-frequency deep TMS^11^ in BD,^16,17^ the data regarding novel protocols such as iTBS and continuous TBS have been inconclusive.^16,24^ Indeed, there is a risk of extending data from MDD dominant samples to BD when novel protocols with unique physiological effects, such iTBS, garner evidence for efficacy in MDD. Our negative RCT highlights the importance of testing the efficacy of novel protocol explicitly in BD.

The 16.7% rate of clinical response in our active iTBS condition is substantially lower than what has been reported in MDD samples treated with iTBS, where response rates typically exceed 40%.^21,22^ The only other iTBS RCT in treating MDE of BD reported rates of clinical response in excess of 70%.^24^ Although this high rate of clinical response is difficult to reconcile with our data, it may be attributable to our eligibility requirement that participants have not responded to a first-line CANMAT-ISBD treatment for bipolar depression. Indeed, the rate of clinical response we observed is in line with a double-blind, sham-controlled trial of bilateral rTMS for bipolar depression where participants were similarly resistant to pharmacotherapy.^36^ It is important to note that our open-label rate of clinical response was also low (23.8%), which suggests that our results are not attributable to inadequate number of treatments during the double-blind phase. In light of growing evidence that rTMS is less effective in bipolar depression compared with MDD,^37,38^ our data suggest that iTBS may also be less effective in bipolar depression than unipolar depression.

Antidepressant treatments in BD carry an increased risk of treatment-emergent mood switches that occur with neurostimulation^39^ and pharmacological interventions,^40^ although not with adequate mood stabilization when used short term.^41^ Case reports suggest that iTBS can induce mania or hypomania in patients with BD,^42^ and, therefore, our design required an antimanic dose. In our sample, the rate of treatment-emergent mood switches was low, yet the only treatment-emergent mood switches occurred in the active iTBS group after the first double-blind treatment and within 6 treatments after transitioning from sham iTBS to open-label treatment. The possibility of treatment emergent mood switches with iTBS in bipolar depression cannot be discounted.

### Limitations

This study is limited by a modest sample size; however, this is the direct consequence of premature termination after interim analyses indicated futility. There were site differences in blinding integrity and the nature of the sham coil; however, this was associated with improvement in depressive symptoms and is unlikely to reflect participant unblinding. Moreover, we included site in all analyses to control for any site-specific effects. Previous data suggest that divalproate and other mainstays of BD treatment can influence motor thresholds.^43^ Whether this impacts clinical outcomes remains to be confirmed; however, our data do not suggest poorer outcomes when patients are treated with valproate or with other mood stabilizers.

Our protocol did not use emerging functional magnetic resonance imaging methods of localizing the LDLPFC target based on connectivity,^17^ and, therefore, it is possible that improved localization may alter clinical outcomes. New data suggest that specific targets are best suited for symptom profiles,^44^ which is something our design did not account for. We also used the stimulation parameters shown to be noninferior to high-frequency stimulation of the LDLPFC in MDD^22^; however, these parameters involve a higher stimulation intensity than what has been efficacious in sham-controlled RCTs in MDD.^21^ As a result, we cannot exclude the possibility that iTBS delivered with other parameters may have different clinical results. It is also unclear whether bipolar depression should be treated using the same targets and stimulation parameters as patients with MDD. This requires dedicated study in individuals with bipolar depression.

## Conclusions

iTBS targeting the LDLPFC does not appear to be clinically efficacious in the treatment of bipolar depression in conjunction with an antimanic agent. Although iTBS does not appear to be associated with increases in manic symptoms in general, we cannot eliminate the possibility that it may result in increased risk of treatment-emergent affective switches. This negative RCT highlights the importance of standardizing protocols and testing the efficacy of neurostimulation treatments proven in MDD in BD. Furthermore, standardizing protocols and sham-controlled designs to account for spontaneous response is necessary to determine what, if any, role TMS has in the treatment of bipolar depression.

## Notes

Supplement 1. **Trial Protocol**

Supplement 2. **Data Sharing Statement**

## References

[1] Blanco C, Compton WM, Saha TD, . Epidemiology of DSM-5 bipolar I disorder: results from the National Epidemiologic Survey on Alcohol and Related Conditions–III. J Psychiatr Res. 2017;84:310-317. doi:10.1016/j.jpsychires.2016.10.003 27814503 PMC7416534

[2] Mcdonald KC, Bulloch AG, Duffy A, . Prevalence of bipolar I and II disorder in Canada. Can J Psychiatry. 2015;60(3):151-156. doi:10.1177/070674371506000310 25886691 PMC4394715

[3] Merikangas KR, Cui L, Kattan G, Carlson GA, Youngstrom EA, Angst J. Mania with and without depression in a community sample of US adolescents. Arch Gen Psychiatry. 2012;69(9):943-951. doi:10.1001/archgenpsychiatry.2012.38 22566563 PMC11955849

[4] Judd LL, Schettler PJ, Solomon DA, . Psychosocial disability and work role function compared across the long-term course of bipolar I, bipolar II and unipolar major depressive disorders. J Affect Disord. 2008;108(1-2):49-58. doi:10.1016/j.jad.2007.06.014 18006071

[5] Kupka RW, Altshuler LL, Nolen WA, . Three times more days depressed than manic or hypomanic in both bipolar I and bipolar II disorder. Bipolar Disord. 2007;9(5):531-535. doi:10.1111/j.1399-5618.2007.00467.x 17680925

[6] Forte A, Baldessarini RJ, Tondo L, Vázquez GH, Pompili M, Girardi P. Long-term morbidity in bipolar-I, bipolar-II, and unipolar major depressive disorders. J Affect Disord. 2015;178:71-78. doi:10.1016/j.jad.2015.02.011 25797049

[7] Kessler RC, Akiskal HS, Ames M, . Prevalence and effects of mood disorders on work performance in a nationally representative sample of U.S. workers. Am J Psychiatry. 2006;163(9):1561-1568. doi:10.1176/ajp.2006.163.9.1561 16946181 PMC1924724

[8] Yatham LN, Kennedy SH, Parikh SV, . Canadian Network for Mood and Anxiety Treatments (CANMAT) and International Society for Bipolar Disorders (ISBD) 2018 guidelines for the management of patients with bipolar disorder. Bipolar Disord. 2018;20(2):97-170. doi:10.1111/bdi.12609 29536616 PMC5947163

[9] Milev RV, Giacobbe P, Kennedy SH, ; CANMAT Depression Work Group. Canadian Network for Mood and Anxiety Treatments (CANMAT) 2016 clinical guidelines for the management of adults with major depressive disorder: section 4—neurostimulation treatments. Can J Psychiatry. 2016;61(9):561-575. doi:10.1177/0706743716660033 27486154 PMC4994792

[10] Brunoni AR, Chaimani A, Moffa AH, . Repetitive transcranial magnetic stimulation for the acute treatment of major depressive episodes: a systematic review with network meta-analysis. JAMA Psychiatry. 2017;74(2):143-152. doi:10.1001/jamapsychiatry.2016.3644 28030740

[11] Tavares DF, Myczkowski ML, Alberto RL, . Treatment of bipolar depression with deep TMS: results from a double-blind, randomized, parallel group, sham-controlled clinical trial. Neuropsychopharmacology. 2017;42(13):2593-2601. doi:10.1038/npp.2017.26 28145409 PMC5686495

[12] Dolberg OT, Dannon PN, Schreiber S, Grunhaus L. Transcranial magnetic stimulation in patients with bipolar depression: a double blind, controlled study. Bipolar Disord. 2002;4(1)(suppl):94-95. doi:10.1034/j.1399-5618.4.s1.41.x 12479689

[13] Nahas Z, Kozel FA, Li X, Anderson B, George MS. Left prefrontal transcranial magnetic stimulation (TMS) treatment of depression in bipolar affective disorder: a pilot study of acute safety and efficacy. Bipolar Disord. 2003;5(1):40-47. doi:10.1034/j.1399-5618.2003.00011.x 12656937

[14] Beynel L, Chauvin A, Guyader N, . What saccadic eye movements tell us about TMS-induced neuromodulation of the DLPFC and mood changes: a pilot study in bipolar disorders. Front Integr Neurosci. 2014;8:65. doi:10.3389/fnint.2014.00065 25191234 PMC4137451

[15] Tamas RL, Menkes D, El-Mallakh RS. Stimulating research: a prospective, randomized, double-blind, sham-controlled study of slow transcranial magnetic stimulation in depressed bipolar patients. J Neuropsychiatry Clin Neurosci. 2007;19(2):198-199. doi:10.1176/jnp.2007.19.2.198 17431073

[16] McGirr A, Karmani S, Arsappa R, . Clinical efficacy and safety of repetitive transcranial magnetic stimulation in acute bipolar depression. World Psychiatry. 2016;15(1):85-86. doi:10.1002/wps.20300 26833619 PMC4780310

[17] Nguyen TD, Hieronymus F, Lorentzen R, McGirr A, Østergaard SD. The efficacy of repetitive transcranial magnetic stimulation (rTMS) for bipolar depression: a systematic review and meta-analysis. J Affect Disord. 2021;279:250-255. doi:10.1016/j.jad.2020.10.013 33074144

[18] Berlim MT, McGirr A, Rodrigues Dos Santos N, Tremblay S, Martins R. Efficacy of theta burst stimulation (TBS) for major depression: an exploratory meta-analysis of randomized and sham-controlled trials. J Psychiatr Res. 2017;90:102-109. doi:10.1016/j.jpsychires.2017.02.015 28254709

[19] Huang YZ, Edwards MJ, Rounis E, Bhatia KP, Rothwell JC. Theta burst stimulation of the human motor cortex. Neuron. 2005;45(2):201-206. doi:10.1016/j.neuron.2004.12.033 15664172

[20] Huang YZ, Chen RS, Rothwell JC, Wen HY. The after-effect of human theta burst stimulation is NMDA receptor dependent. Clin Neurophysiol. 2007;118(5):1028-1032. doi:10.1016/j.clinph.2007.01.021 17368094

[21] Li CT, Chen MH, Juan CH, . Efficacy of prefrontal theta-burst stimulation in refractory depression: a randomized sham-controlled study. Brain. 2014;137(pt 7):2088-2098. doi:10.1093/brain/awu109 24817188

[22] Blumberger DM, Vila-Rodriguez F, Thorpe KE, . Effectiveness of theta burst versus high-frequency repetitive transcranial magnetic stimulation in patients with depression (THREE-D): a randomised non-inferiority trial. Lancet. 2018;391(10131):1683-1692. doi:10.1016/S0140-6736(18)30295-2 29726344

[23] Di Lazzaro V, Dileone M, Pilato F, . Modulation of motor cortex neuronal networks by rTMS: comparison of local and remote effects of six different protocols of stimulation. J Neurophysiol. 2011;105(5):2150-2156. doi:10.1152/jn.00781.2010 21346213

[24] Bulteau S, Beynel L, Marendaz C, . Twice-daily neuronavigated intermittent theta burst stimulation for bipolar depression: a randomized sham-controlled pilot study. Neurophysiol Clin. 2019;49(5):371-375. doi:10.1016/j.neucli.2019.10.002 31761447

[25] Hamilton M. A rating scale for depression. J Neurol Neurosurg Psychiatry. 1960;23:56-62. doi:10.1136/jnnp.23.1.56 14399272 PMC495331

[26] Beam W, Borckardt JJ, Reeves ST, George MS. An efficient and accurate new method for locating the F3 position for prefrontal TMS applications. Brain Stimul. 2009;2(1):50-54. doi:10.1016/j.brs.2008.09.006 20539835 PMC2882797

[27] Montgomery SA, Asberg M. A new depression scale designed to be sensitive to change. Br J Psychiatry. 1979;134:382-389. doi:10.1192/bjp.134.4.382 444788

[28] Young RC, Biggs JT, Ziegler VE, Meyer DA. A rating scale for mania: reliability, validity and sensitivity. Br J Psychiatry. 1978;133:429-435. doi:10.1192/bjp.133.5.429 728692

[29] Guy W. Clinical Global Impressions: ECDEU Assessment Manual for Psychopharmacology–Revised. US Department of Health, Education, and Welfare; 1976.

[30] Broadbent E, Petrie KJ, Main J, Weinman J. The brief illness perception questionnaire. J Psychosom Res. 2006;60(6):631-637. doi:10.1016/j.jpsychores.2005.10.020 16731240

[31] Leon AC, Olfson M, Portera L, Farber L, Sheehan DV. Assessing psychiatric impairment in primary care with the Sheehan Disability Scale. Int J Psychiatry Med. 1997;27(2):93-105. doi:10.2190/T8EM-C8YH-373N-1UWD 9565717

[32] Michalak EE, Murray G; Collaborative RESearch Team to Study Psychosocial Issues in Bipolar Disorder (CREST.BD). Development of the QoL.BD: a disorder-specific scale to assess quality of life in bipolar disorder. Bipolar Disord. 2010;12(7):727-740. doi:10.1111/j.1399-5618.2010.00865.x 21040290

[33] Rush AJ, Trivedi MH, Ibrahim HM, . The 16-Item Quick Inventory of Depressive Symptomatology (QIDS), clinician rating (QIDS-C), and self-report (QIDS-SR): a psychometric evaluation in patients with chronic major depression. Biol Psychiatry. 2003;54(5):573-583. doi:10.1016/S0006-3223(02)01866-8 12946886

[34] Spitzer RL, Kroenke K, Williams JB, Löwe B. A brief measure for assessing generalized anxiety disorder: the GAD-7. Arch Intern Med. 2006;166(10):1092-1097. doi:10.1001/archinte.166.10.1092 16717171

[35] Lam RW, Michalak EE, Swinson RP. Assessment Scales in Depression, Mania and Anxiety. Taylor and Francis; 2005.

[36] Fitzgerald PB, Hoy KE, Elliot D, McQueen S, Wambeek LE, Daskalakis ZJ. A negative double-blind controlled trial of sequential bilateral rTMS in the treatment of bipolar depression. J Affect Disord. 2016;198:158-162. doi:10.1016/j.jad.2016.03.052 27016659

[37] Rostami R, Kazemi R, Nitsche MA, Gholipour F, Salehinejad MA. Clinical and demographic predictors of response to rTMS treatment in unipolar and bipolar depressive disorders. Clin Neurophysiol. 2017;128(10):1961-1970. doi:10.1016/j.clinph.2017.07.395 28829979

[38] Yang YB, Chan P, Rayani K, McGirr A. Comparative effectiveness of repetitive transcranial magnetic stimulation in unipolar and bipolar depression. Can J Psychiatry. Published online August 20, 2020. doi:10.1177/0706743720950938 32815380 PMC7958194

[39] Angst J, Angst K, Baruffol I, Meinherz-Surbeck R. ECT-induced and drug-induced hypomania. Convuls Ther. 1992;8(3):179-185. 11941168

[40] Pacchiarotti I, Bond DJ, Baldessarini RJ, . The International Society for Bipolar Disorders (ISBD) task force report on antidepressant use in bipolar disorders. Am J Psychiatry. 2013;170(11):1249-1262. doi:10.1176/appi.ajp.2013.13020185 24030475 PMC4091043

[41] McGirr A, Vöhringer PA, Ghaemi SN, Lam RW, Yatham LN. Safety and efficacy of adjunctive second-generation antidepressant therapy with a mood stabiliser or an atypical antipsychotic in acute bipolar depression: a systematic review and meta-analysis of randomised placebo-controlled trials. Lancet Psychiatry. 2016;3(12):1138-1146. doi:10.1016/S2215-0366(16)30264-4 28100425

[42] Kaster TS, Knyahnytska Y, Noda Y, Downar J, Daskalakis ZJ, Blumberger DM. Treatment-emergent mania with psychosis in bipolar depression with left intermittent theta-burst rTMS. Brain Stimul. 2020;13(3):705-706. doi:10.1016/j.brs.2020.02.018 32289701

[43] Ziemann U, Reis J, Schwenkreis P, . TMS and drugs revisited 2014. Clin Neurophysiol. 2015;126(10):1847-1868. doi:10.1016/j.clinph.2014.08.028 25534482

[44] Siddiqi SH, Taylor SF, Cooke D, Pascual-Leone A, George MS, Fox MD. Distinct symptom-specific treatment targets for circuit-based neuromodulation. Am J Psychiatry. 2020;177(5):435-446. doi:10.1176/appi.ajp.2019.19090915 32160765 PMC8396109

