# Supplementary material for: Efficacy of Active vs Sham Intermittent Theta Burst Transcranial Magnetic Stimulation for Patients With Bipolar Depression: A Randomized Clinical Trial
Source: JAMA Netw Open. 2021 Mar 12;4(3):e210963. doi: 10.1001/jamanetworkopen.2021.0963 (PMC7955269; doi:10.1001/jamanetworkopen.2021.0963)
Supplement: Supplement 1. — Trial Protocol [file jamanetwopen-e210963-s001.pdf]

A Randomized Double-blind Sham-controlled Trial of Repetitive Transcranial Magnetic Stimulation (rTMS) in Acute Bipolar Depression

18 December 2018

Principal Investigator

Dr. Lakshmi Yatham, MBBS, FRCPC, MRCPsych (UK), MBA (Exec)
Regional Head and Program Medical Director, Mental Health and Addictions, Vancouver Coastal Health and Providence Health Care
Professor, Department of Psychiatry, University of British Columbia
Mood Disorders Clinic- Centre for Brain Health
2215 Wesbrook Mall
Vancouver, British Columbia
V6T 2B5, Canada

Phone: (604) 822-0562
Email: yatham@mail.ubc.ca

Co-Investigators

Raymond W Lam, MD FRCPC
Professor and Associate Head for Research
Department of Psychiatry,
Director, Mood Disorders Centre,
University of British Columbia

Executive Chair, Canadian Network for Mood and Anxiety Treatments (CANMAT)

Phone: 604.822.7325

Alexander McGirr, MD MSc
Psychiatry Resident
Department of Psychiatry
University of British Columbia

Fidel Vila-Rodriguez, MD, FRCPC, FAPA
Clinical Associate Professor
Department of Psychiatry
University of British Columbia

Phone: 604.827.0175

Ivan Torres, PhD
Associate Professor
Department of Psychiatry
University of British Columbia

Phone: 604.827.7769

CONFIDENTIAL AND PROPRIETARY
The contents of this document are confidential and proprietary to The Department of Psychiatry, UBC. Distribution to third parties without prior permission is prohibited.

1. LAY ABSTRACT

Bipolar Disorder is a common condition that is characterized by periods of mood elevation (mania/hypomania), however periods of depression are more common and severely disabling. Effective treatments exist, however many patients do not experience any benefit from them. Hence novel treatment approaches are required for such patients. Neuromodulation techniques involve selective targeting of brain areas which are promising avenues for such bipolar depressed patients.

Repetitive Transcranial Magnetic Stimulation (rTMS) is an effective neuromodulatory technique that is effective in major depression. There are preliminary evidences suggesting that it is effective in bipolar depression as well, however this comes from a number of very small studies and is therefore unclear. Another encouraging finding from these very small studies is that rTMS does not appear to increase the risk of switch to mania; however this is also limited by studies involving only small number of patients.

Given the promising preliminary evidence, we propose to study the efficacy of rTMS in bipolar depression. We will do so using a randomized design in which patients and clinicians will not know whether the patient is receiving daily active iTBS-rTMS or sham-rTMS for four weeks. Our focus is on clinical improvement in depressive symptoms; however we will also look at other aspects such as cognition.

2. STUDY OVERVIEW

**Title**	A randomized double-blind sham-controlled trial of repetitive Transcranial Magnetic Stimulation (rTMS) for acute Bipolar Depression.
**Objectives**	To evaluate the efficacy of intermittent Theta-Burst Stimulation rTMS (iTBS) to the left dorsolateral prefrontal cortex (DLPFC) in treating acute Bipolar Depression.
**Outcome measures**	The primary efficacy measures will be improvement in acute Bipolar Depression as measured by change in Montgomery Asberg Depression Rating Scale (MADRS) with iTBS-rTMS as compared to sham treatment group. In addition we will also examine the improvement in other clinical outcome measures, quality of life and cognition with iTBS-rTMS treatment as compared to sham treatment.
**Study population**	Participants aged 18-70 yrs meeting the Diagnostic and Statistical Manual of Mental Disorder, 5th Edition (DSM-5) criteria for Bipolar diagnosis with a current depressive episode of at least moderate severity will be included.
**Study design**	A prospective, randomized double-blind sham-controlled trial of iTBS-rTMS to the left dorsolateral prefrontal cortex. Participants will complete a screen visit to determine eligibility based on the inclusion/exclusion criteria. If the participants are not eligible, no further study procedures will be conducted. Eligible subjects will be randomized to receive either active iTBS-rTMS or sham rTMS treatment (scalp stimulation with no magnetic pulse) daily for four weeks (20 sessions). Randomization will be computer generated, double-blind random assignment and allocation concealment. All participants will complete a MRI (to target the left DLPFC region of the brain and measure the functional activity), EEG & fNIRS, lab work, and neurocognitive testing prior to the commencement and post rTMS treatment. Clinical lab work will not be conducted at the final visit. Efficacy, safety and tolerability will be evaluated during daily rTMS treatment and during the clinic visits at screen, baseline, Week 2 and post rTMS treatment.<br><br>Non-responders, whether they received active iTBS-rTMS or sham rTMS for 4 weeks will be offered 4 additional weeks of open-label active iTBS-rTMS treatment. The initial blind will be maintained though out the open label phase.<br><br>All participants will have a phone interview two weeks after the last rTMS treatment.
**Sample Size**	We propose a sample size of 100 patients.
**Rating measures**	C**<u>linical Evaluation</u>**:<br>The Mini International Neuropsychiatric Interview (MINI)-7 will be administered to confirm diagnosis of Bipolar disorder. A score $\geq$ 18 on Hamilton Rating Scale for Depression (HAMD) and a score of $\leq$ 8 on Young Mania Rating Scale (YMRS) will be used to define depression. Additional clinical rating scales and self-report measures will be completed.<br>**<u>Cognitive functioning measures</u>**:<br>We will use ISBD-BANC for neurocognitive assessment. Tests that are part of this battery have been shown to be sensitive to identifying deficits in patients with bipolar disorder.
**Efficacy assessments**	The primary efficacy outcome is improvement in depressive symptoms as measured by MADRS scale score from baseline to endpoint. Secondary outcomes are clinical response, clinical remission as well as quality of life and neurocognitive function.
**Safety**	Bipolar disorder specific safety events are the induction of mania/hypomania which will be monitored with the Young Mania Rating Scale.<br><br>The occurrence of any adverse events (seizure, psychosis, confusion, etc) will be recorded and the patient removed from the study if required.

3. LIST OF ABBREVIATIONS

AE	Adverse Events
ATHF	Antidepressant Treatment History Form
BACS	Brief Assessment of Cognition in Schizophrenia
BD	Bipolar Disorder
BIPQ	Brief Illness Perception Questionnaire
CGI-BP-C	Clinical Global Impressions Scale, Bipolar Version, Change
CGI-BP-S	Clinical Global Impressions Scale, Bipolar Version, Severity
CFQ	Cognitive functioning questionnaire
CRF	Case report form
CRQ	Comfort Rating Questionnaire
CVLT-II	The California Verbal Learning Test - II
DLPFC	Dorsolateral prefrontal cortex
DSM.5	Diagnostic and Statistical Manual of Mental Disorder, 5th Edition
EC	Ethics Committee
ECT	Electroconvulsive Therapy
EEG	Electroencephalogram
GAD	Generalized Anxiety Disorder
HAMD	Hamilton Rating Scale for Depression
Hcg	Human Chorionic Gonadotropin
fMRI	functional Near Infrared Spectroscopy - Magnetic Resonance Imaging
ICH-GCP	International Conference on Harmonisation Guideline for Good Clinical Practice
iTBS	Intermittent Theta-Burst Stimulation rTMS
rTMS	repetitive Transcranial Magnetic Stimulation
IRB	Institutional Review Board
ISBD-BANC	The International Society for Bipolar Disorders–Battery for Assessment of Neurocognition
LEAPS	Lam Employment Absence and Productivity Scale
MADRS	Montgomery Asberg Depression Rating Scale
MATRICS	Measurement and Treatment Research to Improve Cognition in Schizophrenia
MDD	Major Depressive Disorder
MINI	Mini International Neuropsychiatric Interview
MRI	Magnetic Resonance Imaging
NAART	North American Adult Reading Test
PGI - S	Patient Global Impression Scale - Severity
PGI - I	Patient Global Impression Scale - Improvement
SAE	Serious Adverse Event
SD	Standard Deviation
SDS	Sheehan Disability Scale
QIDS-SR	Quick Inventory of Depressive Symptomatology-Self Rated
QoL.BD	The Quality of Life in Bipolar Disorder Scale
VAS	Visual Analog Scale
YMRS	Young Mania Rating Scale

29

4. INTRODUCTION

Bipolar disorder is a common condition and a leading cause of global disability [1]. Though characterized by episodes of mania/hypomania, individuals with bipolar disorder suffer a significant portion of their lives with chronic and recurring depressive episodes [2,3]. Indeed, patients are reported to experience symptomatic periods as much as half of their lives with syndromal/sub-syndromal depressive symptoms outnumbering manic/hypomanic symptoms in frequency by a 3:1 ratio [2]. While direct and indirect costs of bipolar disorder are estimated to exceed 70 billion dollars annually in the USA alone [4], the single greatest cost to society is the tragic loss of life associated with suicide in this population [5], which tends to be more common during depressive periods.

Regrettably, few proven treatments exist for bipolar depression despite the tremendous burden[6]. Indeed, quetiapine, fluoxetine plus olanzapine, and lurasidone alone or in conjunction with lithium or valproate are the only FDA approved treatments for acute bipolar depression. Despite limited evidence for efficacy, other pharmacological treatments such as mood stabilizers, other atypical antipsychotics, adjunctive antidepressants and stimulants are also used widely. Antidepressants are widely used in conjunction with mood stabilizers despite continued controversy about their efficacy and safety[7-9]. While expert opinion suggests this is safe when following ISBD recommendations of concomitant mood stabilization [10], their clinical benefit appears to be only modest at the best [11]. Thus, a significant portion of bipolar depressed patients do not respond to or have difficulty tolerating many of these interventions. Adjunctive psychotherapy is beneficial but seldom sufficient for many patients (Parikh et al 2012).

Thus, novel, safe and effective treatments are urgently needed. Though electroconvulsive therapy (ECT) has long been available and effective, some patients are reluctant to consider ECT due to cognitive side effects and stigma. The neuromodulation technique repetitive transcranial magnetic stimulation (rTMS) is a non-invasive, safe and more acceptable method of stimulating brain parenchyma [12]. This technique is not accompanied with the stigma associated with electroconvulsive therapy, and therefore due to its overall acceptability to patients, rTMS has emerged as a viable treatment option.

The most common rTMS approaches are to target either the right or left dorsolateral prefrontal cortex (DLPFC), with either high-frequency (≥5Hz) or low-frequency (1Hz) trains, respectively. Indeed, when utilized in the major depressive disorder (MDD) population, rTMS has demonstrated antidepressant efficacy in numerous double-blind sham-controlled trials, and meta-analyses have demonstrated benefit for high-frequency [13], low-frequency [14], and bilateral DLPFC stimulation paradigms [15]. More recently, the promise of patterned stimulation, most notably theta-burst stimulation, has garnered preliminary evidence for efficacy [16].

While rTMS is efficacious in major depression, it is unclear whether this extends to bipolar depression. Its promise and potential has been highlighted [17], yet the evidence base remains scant. Though open label trials suggest clinical effectiveness[18,19], there have been few dedicated randomized sham-controlled trials [20-23] in the bipolar depressed patient population.

We recently systematically reviewed the literature to identify even small numbers of patients with bipolar disorder included in randomized controlled trials in depression. We were able to identify a total of 181 patients with bipolar depression that had been included in these trials [24]. We found that rTMS was efficacious; however the conclusion is limited because it was based on inclusion of a small number of patients distributed across several trials using different methods and parameters. Thus, the efficacy of rTMS in bipolar depression needs to be assessed in larger randomized controlled trials.

5. OBEJECTIVE

The objective of the proposed study is to determine the efficacy of rTMS directed to the left dorsolateral prefrontal cortex (DLPFC) in acute Bipolar depression. We propose to study active-iTBS-rTMS and sham-rTMS delivered to the DLPFC using a randomized double-blind, sham-controlled design with allocation concealment. Patients with Bipolar Disorder with an acute episode of major depression who have not responded to adequate trials with one of the first line treatments outlined in CANMAT guidelines (i.e. lithium, quetiapine, lamotrigine, lurasidone, fluoxetine plus olanzapine or mood stabilizer plus SSRI or bupropion or lurasidone) will be randomized to receive active iTBS-rTMS or sham-rTMS treatment daily for four weeks (20 sessions).

At the conclusion of the blinded phase, any patient that has not responded to the treatment arm to which they were randomized, (response defined as ≥50% improvement in clinician rated depressive symptoms), will be offered 4 weeks of active-iTBS rTMS treatment.

The primary outcome measures will be improvement in acute bipolar depression as measured by change in Montgomery Asberg Depression Rating Scale (MADRS) with iTBS-rTMS as compared to sham treatment group. In addition we will also be looking at the improvement of clinical outcome measures, quality of life and cognition with iTBS-rTMS treatment as compared to sham treatment.

6. HYPOTHESIS

6.1 Primary Hypothesis

6.1.1 ***Primary efficacy hypothesis***

- Active iTBS-rTMS will result in greater improvements in depressive symptoms as measured with MADRS score compared to sham-rTMS in patients with acute bipolar depression.
-

6.1.2 ***Primary safety hypothesis:***

- Active iTBS-rTMS will not result in higher rates of treatment-emergent affective switch over the course of rTMS treatment.

6.2 Secondary Hypothesis

6.2.1 ***Secondary efficacy hypothesis:***

- Compared to sham-rTMS, active-iTBS rTMS will lead to:
  - Higher rates of clinical remission (score ≤12 on the MADRS) in patients with acute bipolar depression.
  - Higher rates of clinical response (≥50% reduction in MADRS scores) in patients with acute bipolar depression.
  - Greater improvements in quality of life.
  - Greater improvements in neurocognitive function.

6.2.2 ***Secondary safety hypothesis:***

- Compared to sham-rTMS, active-iTBS rTMS in acute bipolar depression will not lead to higher rates of seizure.
- Active iTBS rTMS will not be associated with more side effects than sham-rTMS.

7. STUDY OVERVIEW

The study is a randomized double-blind sham-controlled trial of rTMS to the left dorsolateral prefrontal cortex in acute Bipolar patients. After preliminary eligibility screen based on demographic and clinical information obtained, patients will be approached and a full description of the study will be provided. Participants who are interested will then be provided with informed consent, at which point a full characterization of inclusion and exclusion criteria will be completed. All subjects will be assessed by a study clinician with a clinical interview as well as a MINI neuropsychiatric interview to confirm the diagnosis. The treatment history will be reviewed in order to confirm the refractoriness to at least one of the first line treatments outlined in the updated CANMAT guidelines. Prior to initiating rTMS treatment, all subjects will have a MRI to target the left DLPFC and fMRI, EEG and NIRS to study the functional aspects of the brain. Eligible participants will be randomized to receive 4 weeks of either active iTBS-rTMS or sham (scalp stimulation with no magnetic pulse) rTMS treatment once daily on weekdays. In addition, participants will complete cognitive testing prior to the commencement. All participants who have completed rTMS treatment ≥ 15 sessions will complete a second set of MRI, neurocognitive tests, EEG and fNIRS post rTMS treatments. Efficacy, safety and tolerability will be evaluated during daily rTMS treatment and also at screen, baseline, week 2 and post rTMS treatments.

Non-responders, whether they received active- or sham-iTBS for 4 weeks will be offered 4 additional weeks of open-label active iTBS-rTMS. Non-responders who choose to participate in the additional 4 weeks of iTBS-rTMS will have clinical assessments at week-6 and week-8. The initial blind will be maintained.

All participants will have a follow up telephone interview two weeks after their last rTMS treatment.

8. STUDY POPLULATIONS

100 participants diagnosed with Bipolar with an acute depression will be recruited. Participant will be selected from a pool of subjects that have participated in earlier research studies and have consented for future studies with in the Mood Disorders Center at UBC as well as from outpatients and inpatient units of the Mood Disorders Center at UBC. Recruitment will also occur by referrals from other clinics and online. Patients must meet all of the inclusion criteria and none of the exclusion criteria to be randomized into the study.

Participants must meet all of the following inclusion criteria (8.1) and should not have any of exclusion criteria (8.2) to be eligible to participate in the study.

8.1 Inclusion criteria

1. Males and females aged 18 to 70 years
2. are competent to consent to treatment
3. have a Mini-International Neuropsychiatric Interview (MINI) confirmed diagnosis of DSM-5 criteria for Bipolar Disorder with a current episode of at least moderate severity of depression, single or recurrent
4. have failed to achieve a clinical response to an adequate dose of a first line treatment for Bipolar depression or have been unable to tolerate at least one of the first line treatments
5. have a score ≥ 18 on the HAMD-17 item
6. have a Young Mania Rating Scale Score of ≤ 8
7. are currently taking an anti-manic agent: either lithium with a level of 0.6-1.2 mEq/L, valproate with a level of 350 –700μM, or an atypical antipsychotic. Combinations of these medications, or the combination of any of them with lamotrigine 100-400 mg daily are also permitted. Lamotrigine alone is acceptable as a mood stabilizer for bipolar II patients only.

8. have had no change in dose, or initiation of any psychotropic medication in the 2 weeks prior to randomization
9. are able to adhere to the treatment schedule
10. pass the TMS adult safety screening (TASS) questionnaire

8.2 Exclusion Criteria

1. have an alcohol or substance use disorder within the last 3 months
2. have active suicidal ideation (score of 4 ≥ on item 10 of MADRS)
3. are at a significant risk of harm to themselves or others
4. are currently pregnant , breast feeding or plan to become pregnant
5. have a lifetime Mini-International Neuropsychiatric Interview (MINI) diagnosis of other primary psychiatric diagnoses as assessed by a study investigator to be primary and causing greater impairment than Bipolar diagnosis.
6. are currently taking more than 3 antipsychotics.
7. have failed a course of ECT in the current episode. Previous ECT treatment outside of the current episode does not influence inclusion.
8. history of non-response to rTMS treatment.
9. have any significant neurological disorder or insult including, but not limited to: any condition likely to be associated with increased intracranial pressure, space occupying brain lesion, any history of epilepsy, cerebral aneurysm, Parkinson’s disease, Huntington’s chorea, multiple sclerosis, significant head trauma with loss of consciousness for greater than or equal to 5 minutes
10. have concomitant major unstable medical illness, cardiac pacemaker or implanted medication pump
11. have an intracranial implant (e.g., aneurysm clips, shunts, stimulators, cochlear implants, or electrodes) or any other metal object within or near the head, excluding the mouth, that cannot be safely removed
12. If participating in psychotherapy, must have been in stable treatment for at least 3 months prior to entry into the study, with no anticipation of change in the frequency of therapeutic sessions, or the therapeutic focus over the duration of the study
13. have a clinically significant laboratory abnormality, in the opinion of study physician
14. are currently (or in the last 4 weeks) taking lorazepam greater than 2 mg daily (or equivalent) due to the potential to limit rTMS efficacy
15. have a non-correctable clinically significant sensory impairment (i.e., cannot hear well enough to cooperate with interview).
16. have an exclusion criterion for MRI: Those with a history of cranial, thoracic or abdominal surgery, with pacemakers, artificial joints or other metallic implants will be excluded from the MRI scan. Subjects that have agreed to participate in the MRI portion of the study will be pre-screened for any potential metal fragments in the body (particularly in the orbits) if they have had any history of doing metal work or have been involved in use/deployment of ammunitions/explosives, welding, piping etc).

8.3 Randomization and Allocation Concealment

Randomization will be computer generated, double-blind random assignment and allocation concealment which will be maintained throughout the study. Patients will be randomly assigned to receive either active iTBS-rTMS or sham-rTMS treatments.

8.4 Subject identification number

Randomized subjects will be identified by a unique subject number. There will be no replacement of subjects who withdraw from the study.

9. STUDY VISITS

9.1 Screening Visit:

These procedures will be completed a week prior to beginning the trial

- Written informed consent will be obtained.
- All participants will complete a clinical evaluation using the following rating scales, research interviews and laboratory investigations.
    - The diagnosis of Bipolar Disorder (BD) will be confirmed using the Mini International Neuropsychiatric Interview (MINI-7).
    - Current depressive symptoms, suicidal ideation and manic symptoms will be ascertained by clinical interview, using the 17-item Hamilton Rating Scale for Depression(HAMD-17)[25], Montgomery Asberg Depression Rating Scale (MADRS), Young Mania Rating Scale (YMRS)[26]and Clinical Global Impression-bipolar version- severity scale (CGI-BP-S)

    - Brief Illness Perception Questionnaire (BIPQ)[40] ,Quality of Life in Bipolar Disorder Scale (QOL.BD) and Cognitive Functioning Questionnaire will be administered
    - Psychosocial functioning will be assed Sheehan Disability Scale (SDS).
    - Clinical laboratory Investigations will be completed.
    - Information about concomitant medication use will be obtained.
    - A physical examination will be performed.
- Participants who meet all the inclusion criteria and who don't have any exclusion criteria will be randomised to receive either active iTB-rTMS or sham -rTMS
- All participants will be completing MRI, prior to the baseline visit, to target the left DLPFC region in the brain and the functional activity will be measured using fMRI and simultaneous EEG-fNIRS.
- An Actical watch will be worn on the non-dominant wrist of all participants, between screening and baseline until the end of treatment, in order to monitor sleep patterns and overall level of activity.
- Neurocognitive testing will be conducted by a trained administrator using ISBD-BANC which is shown to be sensitive in identifying deficits in patients with bipolar disorder.

9.2 Daily visits for rTMS treatments:

- Participants will have to come in for daily rTMS treatments for four weeks (20 sessions). The visits will occur on weekdays during working hours (9am-4pm). Participants who miss 3 consecutive sessions within a week will be withdrawn.
- Day of Evaluation and Comfort Rating Questionnaires (A & B) will be completed.
- Over all well-being, concomitant medication and adverse events will be reviewed.
- Non-responders, whether they received active iTBS- rTMS or sham rTMS for 4 weeks will be offered 4 additional weeks of open-label iTBS-rTMS treatment.
- Participants who complete a minimum of 2 weeks (10 sessions) in the double blind phase will be eligible to participate in the open label phase if it is decided by the investigator that it is in the best interest of the participant (eg. in case of significant worsening of depression, emergence of suicidal ideation) to switch to the open label phase.

9.3 Clinical assessments and procedures during and post rTMS treatments:

- Participants in both arms will have clinical assessments and vital signs at baseline, Week 2 and post rTMS treatments.
- MADRS, YMRS, Clinical Global Impression - bipolar version- Severity and Change scale, and Patient Global Impression Severity and Improvement Scale will be administered during these assessments.
- All participants will be completing additional self-report questionnaires.

- Participants who had ≥15 rTMS treatments will be completing a second set of MRI, neurocognitive testing, EEG& fNIRS.
- Non-responders participating in the additional rTMS treatments will have clinical assessment at week-6 and week-8.

9.4 Post study telephone follow-up:

- All participants will complete a telephone follow-up two weeks after their last rTMS session to assess any side effects or issues post treatment.
- A final report will be sent to the primary care physician

10. STUDY SPECIFIC PROCEDURES

Study specific procedures are detailed in the following sections

10. 1: Table 1.0- Flow chart for study procedures.

r TMS in Acute Bipolar Depression								
**Procedures**	**Screen visit (one week prior to rTMS treatment)**	**Baseline visit (first day of rTMS session)**	**Daily rTMS visits**	**Week 2 visit**	**Post rTMS visit**	**Additional 4 weeks of rTMS visits**	**Week 6 & 8 visits**	**Final telephone Follow up**
Informed consent, Contact information, Demographics,	X							
Daily Evaluation Questionnaire		X	X	X		X	X	
**Self-rated scales**								
CRQ A & B		X	X	X		X	X	
QIDS_SR; GAD-7; LEAPS; PGI-S/I		X		X	X		X	
BIPQ, QoL, SDS, CFQ	X				X			
VAS		X			X		**Only at week-8**	
**Physician**								
Inclusion/exclusion criteria	X							
MINI-7+ ADHD	X							
HAM-D- 17	X	X						
MADRS, YMRS, CGI-BP-S /&C	X	X		X	X		X	
Psychiatric, Medical and surgical history, Prior treatment responses, Family Psychiatric and medical history, Physical exam	X							
Laboratory investigations	X							
Randomization	X							
MRI	X				X			
rTMS active/ sham		X	X	X		**Only for non-responders****	X	

EEG & fNIRS	X				X			
Neurocognitive battery- ISBD-BANC	X				X			
Vital Signs	X	X		X	X		X	
Over all well-being, Adverse events, Concomitant medications	X	X	X	X	X	X	X	X
Reimbursement	X		X		X			
*** 20 sessions of rTMS active/sham will be administered over 4 weeks during the weekdays | ** active r TMS will be offered to all non-responders from either arm for four additional weeks and the initial blind will be maintained |**								

10.2 Clinical assessments and patient interview

Diagnosis: Diagnosis of Bipolar disorder DSM.5 criteria will be confirmed by psychiatric examination at screening and administration of the Mini- International Neuropsychiatric Interview (MINI) -Version 7.0. The MINI is a brief, structured diagnostic interview that uses distinct diagnostic modules to assess all current and some lifetime diagnoses that form the major Axis I psychiatric disorders.

Chart Review and clinical interview: Patient charts, where available, will be reviewed to quantify clinical variables such as duration of illness, age of onset, history of mood episodes, number of hospitalizations, and current medications and doses.

Clinical scales and questionnaires: Clinical and medical information will be supplemented by patient interviews using standardized probes and forms. A 17-item Hamilton Rating Scale for Depression (HAMD-17) will be used to measure current depressive symptoms and manic symptoms will be captured using Young Mania Rating Scale (YMRS). In addition, Montgomery Asberg Depression Rating Scale (MADRS), Quick Inventory of Depressive Symptomatology-Self Rated (QIDS-SR), Generalized Anxiety Disorder 7-item scale (GAD-7), and Brief Illness Perception Questionnaire (BIPQ) will be administered. Psychosocial functioning will be captured using a Lam Employment Absence and Productivity Scale (LEAPS) and Sheehan Disability Scale (SDS). The visual analog scale (VAS) will be used to capture overall wellbeing.

Concomitant Treatment: Participants will be allowed to remain on their psychotropic medications as along as the doses have been stable for at least 2 weeks prior to entering the double blind phase. No changes to psychotropic medication are allowed during the double blind phase. The only variation in psychotropic medication that will be considered is zopiclone (up to 15mg per day) or an equivalent for insomnia.

Current medications including OTC medications, herbal remedies, homeopathic preparations, and health and dietary supplements taken by the subject within the previous 30 days will be recorded

Participants must not (or in the last 4 weeks) take more than 2 mg daily (or equivalent) of lorazepam due to the potential to limit rTMS efficacy. If participants are unsure they will be asked to discuss with the investigator before starting the treatment.

Vital Sign Measurement: Vital signs will be obtained at screen, baseline visit, week-2 and post rTMS treatments. Blood pressure will be measured after the subject has been supine for 5 minutes.

Actical Watch:
An Actical Watch is an accelerometer device that records motion. The device does not capture any location or other personal data. The device is pre-loaded with participant unique study ID, age, weight and height, but otherwise contains no personal information. After use and collection of data, these data are downloaded to a secured password-protected laptop. The Actical watch will be worn on the non-dominant wrist of all participants between screening and baseline until the end of treatment. The outcome measure of the Actical is to monitor participants sleep patterns and overall level of activity. This will provide information on whether rTMS has an influence on sleep patterns and the timeline of any potential impact. Also, the level of activity will allow monitoring of non-specific effects between responders and non-responders related to daily contact with research staff (i.e. behavioural activation).

Physical Examination: A complete physical examination will be performed at the screen visit. The complete physical examination will consist of an assessment of the following: general appearance, skin, oedema, lymphadenopathy, head and neck, ears, nose, throat, abdomen, respiratory system, cardiovascular system, neurological system and musculoskeletal system.

Laboratory Assessments: Laboratory measures will be obtained including heamatology, serum chemistry, liver and kidney function, thyroid function, cholesterol lipid screen, plasma electrolytes (including calcium and magnesium), quantitative beta Hcg levels (for all females of childbearing age), and serum levels for the mood stabilizers lithium/valproate in patients taking those medications at the screen visit.

10.3 Neuronavigation: Magnetic Resonance Imaging (MRI)

We propose to utilize the advanced technology to facilitate accurate targeting for this purpose. Participants will undergo an MRI of the brain in order to accurately target the left DLPFC. Using a 3T Philips scanner we will obtain brain scans. Patients will undergo a 90-minute MRI procedure consisting of a high-resolution T1 anatomical sequence for coil placement (10 min), a 48 min Single Voxel MRS sequence targeting both right and left hippocampus as well as the right and left prefrontal cortex (approximately 12min for each scan), a 5 min T2 weighted and a 10 min T2* BOLD fMRI sequence during the resting state for assessment of whole-brain functional connectivity.

All participants will complete the MRI procedure prior to the commencement of rTMS and post rTMS treatment. All participants who completed a minimum of ≥15rTMS treatments will also complete MRI procedures post rTMS treatments.

10.4 rTMS Treatments

Participants will receive either daily iTBS- rTMS or sham-rTMS for four weeks (20 rTMS sessions) once daily on weekdays. Prior to the first treatment, each subject's motor threshold (MT) will first be determined using an established method of neuronavigated TMS over the primary motor cortex. At the first visit for rTMS, the study team will construct a map of each individual's brain from the MRI to aim and target the stimulation work properly. Next, a short stimulation procedure will be performed called motor threshold testing to determine the proper strength of the rTMS, by observing the movements of the hand in response to the stimulation. The rTMS team will locate the motor cortex based on the MRI map of the brain, and use single stimulation at a time to try and make participant's right hand move. The whole procedure will be repeated until the technician has determined which strength of stimulation is best for each participant. The whole motor threshold procedure takes about 3 minutes.

The rTMS system being used for this study involves a method of delivering active- iTBS-rTMS and sham-rTMS in a manner in which blinding is preserved at all times during the study. All participants will have electrodes placed on the scalp capable of delivering small currents to reproduce the sensation of iTBS-rTMS in sham-rTMS in addition to a ‘click’. The system has the capability of administering either active iTBS-rTMS or sham-rTMS based on the patient ID and the blinding code which has been entered.

iTBS rTMS treatment :
Treatment consists of 50 Hz bursts, repeated at 5 Hz; 2 s on and 8 s off; 600 pulses per session; total duration of 3 min 9 s. 120% rMT.

Sham rTMS treatment: Sham rTMS involves a click replicating the sound of the magnetic discharge, without any magnetic pulse being delivered.

The Daily Evaluation Questionnaire will be administered before each rTMS session to ensure that the patient can safely receive the treatment. The study doctor reserves the right to refuse treatment for that day if the patient has recently consumed alcohol/street drugs, or is sleep deprived, or for any another factor that would substantially increase the patients risk for seizure.
The Comfort Rating Questionnaire (CRQ) will also be administered daily to assess the participant’s perception and potential side effects during rTMS treatment (CRQ-A) and after treatment (CRQ-B). If the participant failed to fill out the assessment at home, the CRQ-B will be filled out at UBC before the next day’s treatment session.

10.5 Electroencephalogram (EEG) and functional Near Infrared Spectroscopy (fNIRS)
The induction of electrical current in target brain regions aims to modulate its activity and connectivity to other brain regions. This electrophysiological outcome can be measured using quantitative electroencephalography (EEG), a safe non-invasive procedure involving recording of cortical electrical activity through electrodes placed on the scalp.

Patients will undergo a simultaneous EEG-NIRS protocol. We plan to perform electroencephalographic and blood-flow characterization of the effects of iTBS on the left DLPFC using 61 recording sites determined using 1-20 System of Electrode Placement, an electrode cap and sintered Ag-AgCl electrodes (1000 Hz A/D rate; 0.10 Hz high pass, 200 Hz low pass; gain = 10K; nose reference; impedances ≤ 10 kΩ), we will record resting state EEG and NIRS.

Upon entering the laboratory, participants will be seated in a comfortable chair within a sound-attenuated room. To make the EEG recordings, a mesh cap with small sensors embedded in it will be placed on the participant’s head, and a small sensor will be placed above and below their left eye. The skin beneath the sensors will be cleaned, and a gel will be used to allow contact between the scalp and the sensor.

Functional near-infrared spectroscopy will be recorded by a NIRScout 32-32 device (NIRxMedizintechnik GmbH, Berlin, Germany) with 24 LED sources (intensity 5mW/wavelength) and 32 detectors placed on the frontal, temporal and parietal areas of the scalp. Within each area, two sources and three detectors will be placed. The distance between a source and its neighboring detector will be 3 cm. Each source-detector pair at 3 cm distance will form a channel, resulting in five channels per area and 64 channels in total. The emitted light from sources will be with wavelengths 760 and 850 nm, the sampling rate will be 6.25 Hz.

EEG will be recorded during three paradigms. The first is a resting EEG paradigm, in which participants will be asked to sit quietly with their eyes closed for 5 minutes while their brainwaves are being recorded and then for 5 more minutes with their eyes open fixated on a target visual stimulus on a monitor. These data will allow us to

investigate the magnitude of resting brainwave activity within certain frequency ranges at rest (power), and the relationship between different recording sites on the scalp within those frequency ranges (coherence).

The second paradigm is an auditory entrainment paradigm. During this task, participants will listen to a series of clicks delivered over headphones at specific frequencies. These stimuli encourage the brain to synchronize its firing, or entrain, to the sounds. Previous research has shown that entrainment is associated with NMDA-mediated inhibitory function, which sets the pace for network firing. Therefore, this paradigm is an ideal probe of neural network integrity in a study of rTMS stimulation comparisons. Entrainment data will allow us to investigate the magnitude of neural firing at the stimulated frequencies (power) and the consistency of entrainment over time (inter-trial coherence). The third task is a resting state EEG while listening a guided meditation audio recording for 8minutes.

Both EEG and fNIRS will be done for all subjects once prior to the commencement and once post rTMS treatments. Participants who completed a minimum of ≥15rTMS treatments will be completing the EEG and fNIRS post rTMS treatments

10.6 Neurocognitive testing

The International Society for Bipolar Disorders–Battery for Assessment of Neurocognition (ISBD-BANC) will be administered to assess cognitive functioning prior to the first rTMS treatment and after the last rTMS treatment. Many of the cognitive tests used in the ISBD-BANC overlap with the subtests comprising the Measurement and Treatment Research to Improve Cognition in Schizophrenia (MATRICS) Consensus Cognitive Battery (MCCB), and further include more complex verbal learning measures and tests of executive function that are shown to be sensitive to cognitive impairments in Bipolar Disorder.

- **a) Processing Speed**: The Brief Assessment of Cognition in Schizophrenia (BACS): Symbol Coding Test, Trail Making Test–part A (TMT-A), and Animal Naming Fluency Test from MCCB will be administered to assess a patient’s processing speed.
- **b) Attention:** The Continuous Performance Test–Identical Pairs (CPT-IP) from MCCB will be administered to assess a patient’s attention.
- **c) Working Memory**: The Wechsler Memory Scale–3 (WAIS-III) Letter-Number Sequencing (LNS) and Spatial Span from MCCB will be administered to assess a patient’s working memory.
- **d) Verbal Memory**: The California Verbal Learning Test (CVLT-II) will be administered to assess a patient’s verbal memory. The standard form will be used prior to rTMS treatment and an alternate form will be used at post rTMS treatment to minimize practice effects.
- **e) Nonverbal memory**: The Brief Visuospatial Memory Test–Revised (BVMT) from MCCB will be administered to assess a patient’s nonverbal memory.
- **f) Executive Functioning**: The Stroop Test and Trail Making Test–part B (TMT-B) will be administered to assess a patient’s executive functioning.
- **g) Premorbid IQ Test**: North American Adult Reading Test will be administered to assess a patient’s verbal intellectual ability.

10.7 Time Commitment

Commuting requirements for participants will represent the single greatest time commitment. Second to this, clinical assessments will constitute the next most significant component, followed by the rTMS treatment itself. The screening procedures will be spread over a week and will take 3-7 hours depending on the eligibility and the procedures done. This is followed by 4 weeks of rTMS treatments and daily self-report questionnaires, which is estimated to take approximately 15-30 minutes per session including the set up time, therefore a total of 5-10hrs for the 4 weeks. The weekly clinical assessments and self-report questionnaires at baseline and

week-2 estimated to take 40. A second set of MRI, neurocognitive testing, EEG & fNIRS, including clinical assessments post rTMS treatments will take 4.5-5hrs.

10.8Efficacy measures

The primary efficacy outcome is improvement in depressive symptoms as measured by MADRS scale score from baseline to endpoint. Secondary outcomes are clinical response, clinical remission as well as quality of life and neurocognitive function.

10.9 Withdrawal criteria

10.9.1 Reasons for withdrawal

a) Subjects meeting any of the following criteria will be withdrawn from the study:

- Has a serious or intolerable Adverse Event (see section 11.2 on AEs and SAEs).
- Requires a change in current medication that is prohibited by the protocol.
- Does not follow guidelines specified in the protocol.
- Is non-adherent with study protocol (defined as missing 3 consecutive daily sessions of rTMS).
- Withdraws consent.
- Any signs of suicidal ideation during the clinical investigation (a score of 4 or more on iteam-10 of MADRS).

b) Pregnancies:

Subjects who become pregnant during rTMS treatment must immediately be withdrawn from the trial. A report must be submitted to the IRB. Clinicians will follow local and clinical regulatory guidelines for monitoring and documentation of such events.

10.9.2 Handling of Withdrawals

If a subject prematurely withdraws from the study, the reason for withdrawal will be recorded in the CRF. Subjects who withdraw from the study prematurely will be asked to undergo a clinical assessment.

10.9.3 Study termination

The investigator reserves the right to discontinue the study at any time for any reason. Such a termination must be implemented by the investigator, in a time frame that is compatible with the subject's well-being.

11. SAFETY ASSESSMENTS

11.1 rTMS Safety

11.1.1 Common Side Effects

rTMS has a low rate of side effects and any that occur are often tolerable. Nonetheless, some side effects are notable.[30] The most common complaint pertains to tenderness at the site of stimulation and a headache after treatment (28-39% vs 15% in sham-controlled trials). This is typically relieved by simple non-opioid analgesics.

There is a potential for hearing impairment due to the 'click' that is produced by rapid mechanical deformation of the stimulating coil when current is discharged. The intensity of the noise is often underestimated due to its brief nature, and can result in hearing damage. This, however, is prevented by the use of ear plugs.

In both depressed and healthy subjects, there is a small amount of literature on the possibility of inducing manic and hypomanic symptoms. This small risk for inducing manic symptoms with rTMS is similar to that of electroconvulsive therapy. Although this occurs in a small number of patients, it is a possibility which can be partially anticipated in this study design by performing a thorough patient and family history suggestive of any episodes of mania or hypomania.

11.1.2 Treatment Emergent Affective Switch

Treatment of bipolar depression can be associated with the development of mania/hypomania. Though the available literature suggests that this is rare in bipolar disorder (approximately 1% in both active-rTMS and sham-rTMS), it remains a concern. Patients will be carefully monitored and treatment discontinued should they experience manic/hypomanic symptoms.

11.1.3 Seizures

Intrinsic to rTMS is the risk of unintentionally triggering a seizure[30]. Though there have been no published reports of seizure associated with rTMS in Bipolar Disorder, there have been reports in other populations. Several risk factors have been identified, specifically a prior history of epilepsy or medication-induced seizures, or other neurological condition, including prior cerebrovascular accident. In the absence of such predisposing factors, dopaminergic medications have been associated with rTMS seizures, as have stimulation parameters with inadequate inter-stimulation intervals (≤1 second) and stimulation parameters exceeding the motor threshold (such as stimulation intensities as high as 208% of motor threshold).

For this reason, our protocol involves several attempts to reduce the risk of seizure. First, a careful medical history is taken to evaluate past instances of seizure. Second, patients currently taking dopaminergic agents, such as the antidepressant bupropion, will not be enrolled in this study.

A physician will be available on call during all rTMS sessions. Sessions will be filmed and maintained on a temporary (automatically erased daily) USB drive in order to review any adverse event during stimulation.

12. ADVERSE EVENTS

All AEs, whether observed by the investigator, reported by the subject, noted from laboratory findings, or identified by other means, will be recorded from the time the subject signs the ICF until the subject's final study visit. All AEs will be recorded in the source documents.

12.1 Definition

An Adverse Event (AE) is any reaction, side effect, or other untoward event, regardless of relationship to study drug, which occurs any time after the subject signs the ICF until completion of the final study visit or early termination visit. During the study, clinically significant adverse changes in laboratory values (not associated with an AE or concurrent medical condition), and physical examinations are considered AEs.

Adverse events include, but are not limited to: (1) a worsening or change in nature, severity, or frequency of conditions present at the start of the study; (2) subject deterioration due to primary illness; (3) intercurrent illness; and (4) drug interaction.

Subjects should be questioned in a general way, without asking about the occurrence of any specific symptom. The investigator should attempt to establish a diagnosis of the event based on signs, symptoms, and/or other clinical information. In such cases, the diagnosis should be documented as the AE and not the individual signs/symptoms.

Following questioning and evaluation, all AEs, whether believed by the investigator to be related or unrelated to the study drug, will be captured in the subject source documents. Each AE is to be evaluated for duration, intensity, frequency, seriousness, outcome, other actions taken, and relationship to the study treatment. An AE is deemed associated with the use of the study treatment "if there is a reasonable possibility that the AE may have been caused by the treatment" (21 CFR 312.32 [a]). It should be noted that the term "severe" used to grade intensity is not synonymous with the term "serious."

12.1.1 Intensity of Adverse Events

Adverse events must be graded for intensity. An intensity category of mild, moderate, or severe, as defined in Table 2, must be entered on the AE CRF.

Table 2. Intensity of Adverse Events

Grade	Intensity
**Mild**	Causing no limitation of usual activities; the subject may experience slight discomfort.
**Moderate**	Causing some limitation of usual activities; the subject may experience annoying discomfort.
**Severe**	Causing inability to carry out usual activities; the subject may experience intolerable discomfort or pain.

12.1.2 Relationship to Study treatment

The investigator/ study physician will document his/her opinion of the relationship of the AE to treatment using the criteria outlined in Table 3.

Table 3. Relationship of Adverse Events to Study procedure

Relationship	Description
**Definite**	A reaction that follows a reasonable temporal sequence from administration of the study procedure/drug; that follows a known or expected response pattern to the suspected procedure/drug; and that is confirmed by improvement on stopping or reducing number or treatment procedures/dosage of drug , and reappearance of the reaction on repeated exposure
**Probable**	A reaction that follows a reasonable temporal sequence from administration of the study procedure/drug; that follows a known or expected response pattern to the suspected procedure; that is confirmed by stopping or reducing study treatment procedure/ dosage of the drug; and that could not be reasonably explained by the known characteristics of the subject's clinical state.
**Possible**	A reaction that follows a reasonable temporal sequence from administration of the study procedure/drug; that follows a known or expected response pattern to the suspected procedure/drug; but that could readily be produced by a number of other factors.
**Unlikely**	A reaction that follows a reasonable temporal sequence from administration of the study procedure /drug; that follows a known or suspected response pattern to the suspected study procedure/drug; but that could reasonably be explained by known characteristics of the subject's clinical state.
**Not Related**	Any event that does not meet the above criteria.

12.2 Recording Adverse Events

Each AE will be listed as a separate entry on an AE CRF. Screen failure patients will have AE information noted in the source documentation. The investigator will provide information on dates of onset and resolution, intensity, seriousness, action(s) taken, changes in study drug usage, relationship to study procedure or drug, and outcome (if applicable). For randomized subjects, an AE that worsens in intensity will be recorded as a new AE entry in the AE CRF.

12.3 Post-study Follow-Up of Adverse Events

All AEs, including physical examination findings, or isolated clinically significant laboratory findings must be followed until the event resolves, the condition stabilizes, the event is otherwise explained, or the subject is lost to follow-up. If resolved, a resolution date should be documented on the CRF. The investigator is responsible for ensuring that follow-up includes any supplemental investigations as may be indicated to elucidate the nature and/or causality of the AE. This may include additional laboratory tests or investigations, or consultation with other health care professionals, as is practical.

12.4 Serious Adverse Events

All SAEs will be recorded from the time the subject has signed the ICF until 30 days after the last study dose/procedure or final study visit, whichever is longer in duration.

12.4.1 Definition of a Serious Adverse Event

Any untoward medical occurrence which:

- Results in death
- Is life threatening. Life-threatening means that the subject was, in the view of the investigator, at immediate risk of death from the reaction as it occurred at the time of the event; it does not refer to an event that hypothetically might have caused death if it were more severe.
- Requires inpatient hospitalization, or, in the opinion of the investigator, prolongation of existing hospitalization. Hospitalization for elective treatment or a pre-existing condition that did not worsen during the clinical investigation is not considered an AE.
- Results in persistent or significant disability or incapacity
- Is a congenital anomaly or birth defect

12.4.2 Managing Serious Adverse Events

Subjects experiencing an SAE or an emergency situation will be examined by a physician as soon as possible. The physician in attendance will do whatever is medically needed for the safety and well-being of the subject. The subject will remain under observation as long as medically indicated. Appropriate laboratory studies will be conducted until all parameters return to normal or are otherwise explained or stable. The subject will be followed until the SAE resolves or until the subject is medically stabilized. The Principal Investigator or designee will notify the IRB, if necessary immediately (within 24 hours and no later than 7 days) of the SAE and the outcome of the SAE.

If within the time of informed consent until 30 days after the last study dose/procedure, an investigator becomes aware of an SAE, then the event must be documented and reported as described in Section 12.4.3.

12.4.3 Reporting Serious Adverse Events

Serious Adverse Events must be reported within 24 hours of first knowledge of the event by study personnel to the principal investigator and an SAE Form should be completed. It is important that the investigator provide his or her assessment of relationship to study procedure at the time of the initial report.

12.4.4 Conditions for Unblinding, Initiating Experiment 2 and Trial Discontinuation.

Unblinding : Unblinding will happen immediately in the event of a seizure. The participant will immediately be withdrawn from the study.

Trial Discontinuation: The trial will be discontinued if the treatment-emergent affective switches are increased in the active rTMS group compared to the sham-rTMS group ($\alpha \leq 0.1$) at the interim analyses planned after the 20th participant.

13. STATISTICAL ANALYSES AND REPORTING

Reporting of results will occur in aggregate form with no personally identifiable data.

13.1 Power Calculations

Primary hypotheses of change in MADRS scores will be tested using mixed effects model with repeated measures while clinical response and remission will be determined using logistic regression.

- A sample size of 100 patients would allow 0.80 power to detect significance with a medium effect size of ≥ 0.3 with an $\alpha \leq 0.05$.

13.2 Clinical Data

Statistical analyses will be carried out using SPSS v23 (Chicago, IL). Significance will be set at $\alpha \leq .05$. Comparisons of dichotomous outcomes will use Chi-square tests (with 95% Confidence Interval). Baseline comparisons of continuous variables will take place using Student's t-test for normally distributed data and Mann-Whitney test for non-normally distributed variables.

The primary efficacy outcome is improvement in depressive symptoms as measured by MADRS scale score from baseline to endpoint. Secondary outcomes are clinical response, clinical remission as well as quality of life and neurocognitive function.

Analyses for MADRS change scores from baseline to week 4 will be performed using mixed effects model for repeated measures with treatment group and treatment group-by-visit interaction as fixed effects and baseline value and baseline-by-visit interaction as covariates. Response and remission rates will be determined using logistic regression with the last observation carried forward.

13.3 Neuropsychological Data

Statistical analyses will be carried out using SPSS v23 (Chicago, IL). Significance will be set at $\alpha \leq .05$. Repeated measures ANOVA will be employed, and secondary analyses will be performed using repeated measures ANCOVA (controlling for HAMD-17 scores).

13.4 Neuroimaging

Statistical analyses will be carried out using SPSS v23 (Chicago, IL). Significance will be set at $\alpha \leq .05$. Comparisons will be performed as a function of antidepressant response to rTMS using Student's t-test for normally distributed data and Mann-Whitney test for non-normally distributed variables. Structural morphometry will be analyzed using free surfer. Tractography, with stimulation site region of interest, will be performed using Matlab.

13.5. Data Management

In this double blind, randomized, placebo-controlled study, participants will be assigned a study ID that will be a 4-digit code on all the CRF's with no relation to any personal identifiable information. These codes will be linked to their identifiable information only in a locked filing cabinet accessible only to the principal investigator or his designee.

In a separate locked filing cabinet accessible only to the blinding trustee (**Ivan Torres**, PhD, Associate Professor, Mood disorders clinic) will be the master key linking the study ID to the treatment condition. The data will only be unblinded at set interim analyses, at the conclusion of the trial or in the event of a seizure.

All data will be kept in anonymized format, on password protected computers and transferred only using password protected encrypted USB drives. Data will be kept as required by ICH-GCP guidelines.

14. REFERENCES

1. WHO. The global burden of disease: 2004 update. In: Informatics DoHSa, ed. Geneva: WHO Press; 2008.
2. Judd LL, Schettler PJ, Solomon DA, et al. Psychosocial disability and work role function compared across the long-term course of bipolar I, bipolar II and unipolar major depressive disorders. J Affect Disord 2008;108:49-58.
3. Kupka RW, Altshuler LL, Nolen WA, et al. Three times more days depressed than manic or hypomanic in both bipolar I and bipolar II disorder. Bipolar Disord 2007;9:531-5.
4. Dilsaver SC. An estimate of the minimum economic burden of bipolar I and II disorders in the United States: 2009. J Affect Disord 2011;129:79-83.
5. Nordentoft M, Mortensen PB, Pedersen CB. Absolute risk of suicide after first hospital contact in mental disorder. Arch Gen Psychiatry 2011;68:1058-64.
6. Yatham LN, Kennedy SH, Parikh SV, et al. Canadian Network for Mood and Anxiety Treatments (CANMAT) and International Society for Bipolar Disorders (ISBD) collaborative update of CANMAT guidelines for the management of patients with bipolar disorder: update 2013. Bipolar Disord 2013;15:1-44.
7. Ghaemi SN, Rosenquist KJ, Ko JY, Baldassano CF, Kontos NJ, Baldessarini RJ. Antidepressant treatment in bipolar versus unipolar depression. Am J Psychiatry 2004;161:163-5.
8. Gijsman HJ, Geddes JR, Rendell JM, Nolen WA, Goodwin GM. Antidepressants for bipolar depression: a systematic review of randomized, controlled trials. Am J Psychiatry 2004;161:1537-47.
9. Sidor MM, Macqueen GM. Antidepressants for the acute treatment of bipolar depression: a systematic review and meta-analysis. J Clin Psychiatry 2011;72:156-67.
10. Pacchiarotti I, Bond DJ, Baldessarini RJ, et al. The International Society for Bipolar Disorders (ISBD) task force report on antidepressant use in bipolar disorders. Am J Psychiatry 2013;170:1249-62.
11. McGirr A, Vöhringer PA, Ghaemi SN, Yatham LN. The safety and efficacy of adjunctive modern antidepressant therapy with a mood stabilizer or antipsychotic in acute bipolar depression: A meta-analysis of randomized placebo-controlled trials. in review.
12. George MS, Aston-Jones G. Noninvasive techniques for probing neurocircuitry and treating illness: vagus nerve stimulation (VNS), transcranial magnetic stimulation (TMS) and transcranial direct current stimulation (tDCS). Neuropsychopharmacology 2010;35:301-16.
13. Berlim MT, van den Eynde F, Tovar-Perdomo S, Daskalakis ZJ. Response, remission and drop-out rates following high-frequency repetitive transcranial magnetic stimulation (rTMS) for treating major depression: a systematic review and meta-analysis of randomized, double-blind and sham-controlled trials. Psychol Med 2014;44:225-39.
14. Berlim MT, Van den Eynde F, Jeff Daskalakis Z. Clinically Meaningful Efficacy and Acceptability of Low-Frequency Repetitive Transcranial Magnetic Stimulation (rTMS) for Treating Primary Major Depression: A Meta-Analysis of Randomized, Double-Blind and Sham-Controlled Trials. Neuropsychopharmacology 2013;38:543-51.
15. Berlim MT, Van den Eynde F, Daskalakis ZJ. A systematic review and meta-analysis on the efficacy and acceptability of bilateral repetitive transcranial magnetic stimulation (rTMS) for treating major depression. Psychol Med 2013;43:2245-54.
16. Li C-T, Chen M-H, Juan C-H, et al. Efficacy of prefrontal theta-burst stimulation in refractory depression: a randomized sham-controlled study. Brain;137:2088-98.
17. Loo C, Greenberg B, Mitchell P. Nonpharmacotherapeutic Somatic Treatments for Bipolar Disorder (ECT, DBS, rTMS). Curr Top Behav Neurosci 2011;5:285-302.
18. Harel EV, Zangen A, Roth Y, Reti I, Braw Y, Levkovitz Y. H-coil repetitive transcranial magnetic stimulation for the treatment of bipolar depression: an add-on, safety and feasibility study. World J Biol Psychiatry 2011;12:119-26.

19. Dell'Osso B, Mundo E, D'Urso N, et al. Augmentative repetitive navigated transcranial magnetic stimulation (rTMS) in drug-resistant bipolar depression. Bipolar Disord 2009;11:76-81.
20. Dolberg OT, Dannon PN, Schreiber S, Grunhaus L. Transcranial magnetic stimulation in patients with bipolar depression: a double blind, controlled study. Bipolar Disord 2002;4 Suppl 1:94-5.
21. Nahas Z, Kozel FA, Li X, Anderson B, George MS. Left prefrontal transcranial magnetic stimulation (TMS) treatment of depression in bipolar affective disorder: a pilot study of acute safety and efficacy. Bipolar Disord 2003;5:40-7.
22. Beynel L, Chauvin A, Guyader N, et al. What saccadic eye movements tell us about TMS-induced neuromodulation of the DLPFC and mood changes: a pilot study in bipolar disorders. Front Integr Neurosci 2014;8:65.
23. Tamas RL, Menkes D, El-Mallakh RS. Stimulating research: a prospective, randomized, double-blind, sham-controlled study of slow transcranial magnetic stimulation in depressed bipolar patients. J Neuropsychiatry Clin Neurosci 2007;19:198-9.
24. McGirr A, Karmani S, Arsappa R, et al. The clinical efficacy and safety of repetitive transcranial magnetic stimulation (rTMS) in bipolar depression: a systematic review and meta-analysis of patients with bipolar disorder included in randomized, double-blind, and sham-controlled trials. World Psychiatry in press.
25. Hamilton M. A rating scale for depression. Journal of Neurology Neurosurgery and Psychiatry 1960;23:56-62.
26. Young RC, Biggs JT, Ziegler VE, Meyer DA. A rating scale for mania: reliability, validity and sensitivity. Br J Psychiatry 1978;133:429-35.
27. Spitzer RL, Kroenke K, Williams JB, Lowe B. A brief measure for assessing generalized anxiety disorder: the GAD-7. Arch Intern Med 2006;166:1092-7.
28. Rush AJ, Trivedi MH, Ibrahim HM, et al. The 16-Item Quick Inventory of Depressive Symptomatology (QIDS), clinician rating (QIDS-C), and self-report (QIDS-SR): a psychometric evaluation in patients with chronic major depression. Biol Psychiatry 2003;54:573-83.
29. Becking K, Haarman B, van der Lek R, et al. Inflammatory monocyte gene expression: trait or state marker in bipolar disorder? Int J Bipolar Disord 2015;3:20. doi: 10.1186/s40345-015-0037-x. Epub 2015 Sep
30. Loo CK, McFarquhar TF, Mitchell PB. A review of the safety of repetitive transcranial magnetic stimulation as a clinical treatment for depression. Int J Neuropsychopharmacol 2008;11:131-47.
