# Supplementary material for: Efficacy of Active vs Sham Intermittent Theta Burst Transcranial Magnetic Stimulation for Patients With Bipolar Depression: A Randomized Clinical Trial
Source: JAMA Netw Open. 2021 Mar 12;4(3):e210963. doi: 10.1001/jamanetworkopen.2021.0963 (PMC7955269; doi:10.1001/jamanetworkopen.2021.0963)
Supplement: Supplement 2. — Data Sharing Statement [file jamanetwopen-e210963-s002.pdf]

Data Sharing Statement

McGirr. Efficacy of Active vs Sham Intermittent Theta Burst Transcranial Magnetic Stimulation for Patients With Bipolar Depression. *JAMA Netw Open*. Published March 12, 2021.
doi:10.1001/jamanetworkopen.2021.0963

Data
Data available: No

Additional Information
Explanation for why data not available: Individual patient data transfer was not included in our ethics protocol.
